# The Usefulness of Basic Laboratory Analyses in Diagnostics of Inherited Metabolic Diseases in Children

**DOI:** 10.3390/diagnostics15212806

**Published:** 2025-11-05

**Authors:** Patryk Lipiński, Anna Doroba

**Affiliations:** 1 Department of Pediatrics, Bielański Hospital, 01-809 Warsaw, Poland; 2 Department of Pediatrics, Medical Center of Postgraduate Education, 01-813 Warsaw, Poland

**Keywords:** inherited metabolic diseases, selective screening, laboratory analyses, children

## Abstract

Pediatricians play a crucial role in the early diagnosis and management of inherited metabolic diseases (IMDs). The diagnosis of IMD is based on the principle of selective screening, often requiring highly specialized testing, including biochemical and molecular analyses. The aim of this comprehensive review was to provide pediatricians with the usefulness of basic laboratory analyses in diagnostic process of IMD. The abnormal results of basic laboratory analyses were thoroughly discussed in the context of clinical manifestations of IMDs. Easy-to-use algorithms were implemented to ensure an appropriate differential diagnosis.

## 1. Introduction

Inherited metabolic diseases (IMDs), also known as inborn errors of metabolism, constitute a group of genetically determined (mostly monogenic) disorders associated with defects in proteins involved in various biochemical processes (pathways) [1,2,3]. An exact defect in the protein’s structure or its function results from the presence of pathogenic molecular variants (formerly mutations) in protein-coding genes. The current (year 2023) classification of IMDs includes a total number of 1450 disorders divided into 24 categories encompassing 124 groups [4,5].

The dynamics of substrate influx/synthesis vary across different cell and tissue types. Metabolic processes occur in their specific cellular organelles/cytosolic pathways, allowing for the integration of metabolism—not all are equally important in all cell types. The effect of a given defect may therefore be different in the central or peripheral nervous system, macrophages, or liver cells. This manifests itself in characteristic clinical and/or biochemical features, which may be limited primarily to a single cell/tissue type.

The main diagnostic strategies used in the diagnostic process of IMDs include the following: (i) population-based screening (newborn screening); (ii) selective screening (testing after the onset of symptoms); (iii) screening for risk groups (i.e., family screening); (iv) post-mortem testing (autopsy examination) [1,2,3].

The varying expression and severity of symptoms in IMDs, and also the possibility of causative treatment in selected cases, necessitate establishing a diagnosis as early as possible. The diagnosis of IMD is based on the principle of selective screening—if a symptom/cluster of symptoms suggest(s) a (known) metabolic disease, testing for this condition is performed, not necessarily to confirm the suspicion, but to rule it out. Diagnosing an IMD always requires a thorough medical history (including family history, pregnancy, and perinatal history), a detailed physical examination of the patient, and analyzing the results of additional tests in the context of the clinical picture of the disease. Confirming the diagnosis of IMD often requires highly specialized testing, including biochemical and molecular (genetic) analyses. However, the results of basic laboratory tests, combined with the results of the medical history and physical examination, are essential to guide further diagnostics (Table 1). Recently, there has been a trend toward a complete reversal of the diagnostic process, beginning with molecular testing—a shift “from genotype to phenotype” [6]. The implementation of high-throughput next-generation sequencing (NGS) techniques for diagnostic purposes marked such a turning point. It is important to note that not every (genetic) disease can be diagnosed using NGS, and not every NGS test result will yield a definitive diagnosis.

The aim of the manuscript was to provide the pediatricians with the usefulness of basic laboratory analyses in the first step of diagnostics of IMD in children. As pediatricians play a crucial role in diagnosis and management of IMDs, it is imperative that clinicians do not miss early opportunities for diagnosis. The paper constitutes an up-to-date literature review on common findings in laboratory results of IMDs in children enriched with the authors’ personal experience.

## 2. Usefulness of Basic Laboratory Analyses in Diagnosis of IMDs

The following laboratory/biochemical parameters are usually used as the first-line tests in the diagnosis of IMDs: (i) peripheral (complete) blood count; (ii) laboratory (biochemical) markers of liver damage/function, including aminotransferases (also known as transaminases) activity: alanine (ALT *) and aspartate (AST **); it should be noted that serum creatinine kinase (CK ***) activity should always be investigated simultaneously; parameters of liver synthetic function: serum albumin concentration, prothrombin time (PT), and international normalized ratio (INR); parameters of liver secretory function (cholestasis): total and conjugated serum bilirubin concentration, gamma-glutamyltranspeptidase (GGT) activity, serum bile acid concentration; (iii) fasting blood glucose assessment (or 24-h blood glucose profile)—in the event of hypoglycemia ^#^, blood gas analysis, ionogram, ammonia and lactic acid concentration, ketosis assessment (urine alysis, free fatty acids, beta-hydroxybutyric acid), and, in selected cases, assessment of hormonal imbalances (cortisol, ACTH, insulin, C-peptide); (iv) lipid profile—total cholesterol, LDL and HDL cholesterol, triglycerides; (v) assessment of water–electrolyte and acid–base imbalances: blood gas analysis, serum ionogram (sodium, potassium, chloride, calcium, magnesium, and phosphate—the concentration/excretion of selected ions in urine is important in assessing the cause of a given ion deficiency; (vi) laboratory parameters of renal function: serum creatinine, urine analysis, serum uric acid; (vii) repeated (at least twice) measurements (to avoid false-positive results) of serum ammonia ^##^ and lactate concentration—it should be noted that immediate ammonia measurement in an emergency setting is crucial and simultaneous laboratory/biochemical diagnostics and treatment is pivotal.

* Reference ranges for ALT: <18 months of age: ≤60 IU/L, 18 months of age–12 years of age: boys ≤ 40 IU/L, girls ≤ 35 IU/L, >12 years of age: boys ≤ 26 IU/L, girls ≤ 22 IU/L.

** AST activity should be compared to the reference values used by the laboratory.

*** Reference ranges for normal values, depending on the standards used by individual laboratories, are approximately 40–285 U/L for women and 55–370 U/L for men. It is believed that CK activity is significantly influenced by the patient’s age, gender, race, and physical activity.

^#^ Definition of hypoglycemia: blood glucose concentration < 2.6 mmol/L (45 mg/dL) regardless of age.

^##^ Conversion of ammonia concentration: μg/dL × 0.5872 = μmol/L.

## 3. Peripheral Blood Morphology

### 3.1. Megaloblastic Anemia

Megaloblastic anemia due to vitamin B12 (synonym: cobalamin, Cbl) deficiency could be related to nutritional and acquired Cbl deficiencies, and inborn errors of Cbl absorption and intracellular trafficking (Table 2) [7,8,9].

Megaloblastic anemia is often observed in the neonatal forms of intracellular Cbl defects (cblF, cblJ, cblC, cblD-MMA/Hcy, cblD-HC, cblE, and cblG) but less frequently in the late-onset forms of these disorders (Table 2) [8,9]. The clinical presentation of the cblF, cblJ, and combined cblD-MMAHcy defects include feeding difficulties, developmental delay, muscular hypotonia, metabolic crisis with acute encephalopathy, seizures, and acidosis [8,9]. The cblC defect is the most frequent combined remethylation disorder and in addition to the above-mentioned clinical presentation, renal manifestations could be observed (atypical haemolytic uraemic syndrome (HUS), glomerulopathy) [8,9]. The other inherited disorders of cbl metabolism leading to megaloblastic anemia in infancy or early childhood include transcobalamin II deficiency (with failure to thrive and gastrointestinal manifestation), Imerslund–Grasbeck syndrome (with proteinuria), and less commonly gastric intrinsic factor deficiency.

Methylmalonic acid (MMA) in blood is the most sensitive marker of intracellular, functionally relevant Cbl depletion while hyperhomocysteinaemia is the hallmark of the remethylation and transulfuration disorders (Table 2). It should be noted that maternal vitamin B12 deficiency needs to be excluded in neonates and young infants presenting with biochemical features of a remethylation disorder.

IMDs leading to megaloblastic anemia through impaired folate metabolism include methylenetetrahydrofolate dehydrogenase 1—MTFHD1 (catalyzing 5,10-MTHF formation via the synthesis of 10-formyl-THF and 5,10-methenyl-THF), dihydrofolate reductase—DHFR (catalyzing the reduction of dihydrofolate to tetrahydrofolate and, at a lower rate, of folic acid to DHF) and formiminotransferase-cyclodeaminase (FTC) deficiencies [7,8,9]. MTFHD1 deficiency is usually associated with hyperhomocysteinemia, megaloblastic anemia, atypical hemolytic uremic syndrome (HUS), recurrent infections, and autoimmunity [7]. DHFR deficiency causes megaloblastic anemia and cerebral folate deficiency leading to various neurological presentations (developmental delay, epilepsy) [8]. Severe FTC deficiency is characterized by formiminoglutamic (FIGLU) aciduria, megaloblastic anemia, and intellectual disability [9].

### 3.2. Microcytic Anemia

The three main types of diseases leading to microcytic anemia in the course of IMDs are iron transport disorders, sideroblastic anemias, and porphyrias [7,10].

Iron deficiency anemia is the most common type of anemia in children; however, it could co-exist with anemia of chronic disease (which is usually a diagnosis of exclusion).

Anemia is very common among patients with glycogen storage disease type I and is usually multifactorial (iron deficiency, anemia of chronic disease, kidney disease).

The clinical picture of porphyrias associated with microcytic anemia, namely congenital erythropoietic porphyria (CEP) and erythropoietic protoporphyria (EPP), is very characteristic due to an acute painful phototoxicity on sun-exposed skin areas [11,12,13,14].

Sideroblastic anemias are associated with mitochondrial iron accumulation in bone marrow erythroid precursors (ringed sideroblasts), ineffective erythropoiesis, and varying proportions of hypochromic erythrocytes in the peripheral blood [10]. Among IMDs, sideroblastic anemia is observed in Pearson Syndrome (PS), Kearns–Sayre syndrome (see Section 5), and thiamine-responsive megaloblastic anemia.

Anemia could be the sole presenting symptom of PS; however, majority of patients present with neutropenia and thrombocytopenia. Other common initial symptoms include failure to thrive, vomiting, diarrhea, and feeding difficulties. Patients usually require both red blood cell and platelet transfusions; however, a hematological recovery usually occurs at the age of 1 to 3 years [10,15]. Hematological features of PS, such as pancytopenia, elevated HbF and mean corpuscular volume, and marrow hypoplasia and dysplasia resemble those of other inherited BM failure disorders, including Diamond–Blackfan anemia and Shwachman–Diamond syndrome [7,10].

Variants in *YARS2* have been recently associated with myopathy, lactic acidosis, and sideroblastic anemia 2 (MLASA2) [16].

### 3.3. Hemolytic Anemia

Disorders of the red cell membrane and hemoglobinopathies represent the most common cause of inherited hemolytic anemia. Abetalipoproteinemia and sitosterolemia (see Section 11) represent the inherited lipoprotein lipid metabolism disorders associated with hemolytic anemia with acanthocytes and stomatocytes seen on the blood film, respectively. The cblC defect presents with microangiopathic hemolytic anemia (see Section 3.1).

### 3.4. Thrombocytopenia

In every patient presenting with splenomegaly or hepatosplenomegaly, especially with concomitant thrombocytopenia, Gaucher disease (GD) should first be ruled out (due to its higher incidence) and then acid sphingomyelinase deficiency (ASMD) [17]. Thrombocytopenia is frequently observed in patients with chronic neurovisceral and visceral forms of ASMD and results from bone marrow infiltration and hypersplenism.

Accumulation of glucocerebroside in Browicz–Kuppfer cells in GD leads to a significant enlargement of the liver and spleen volume without hepatocyte damage (normal ALT activity). Nosebleeds, gingival bleeding, prolonged menstrual bleeding, and a tendency to bruise easily are a consequence of thrombocytopenia, which occurs in virtually all patients; anemia is less common (in 20–50% of patients); leukopenia is the least common [18].

### 3.5. Neutropenia

Barth syndrome is an X-linked syndrome associated with neutropenia that may be chronic, intermittent, or cyclic [19]. Neutropenia is usually accompanied by motor delay, myopathy/cardiomyopathy, feeding problems, failure to thrive, or suspected mitochondrial disease, although presentation with isolated neutropenia can also occur [19].

Patients with glycogen storage disease type Ib (GSD Ib) usually present in infancy with severe fasting hypoglycemia, hepatomegaly, neutropenia (with severity varying from mild to agranulocytosis), and neutrophil dysfunction [20,21]. Since 2019, the SGLT2-inhibitor empagliflozin has provided a mechanism-based treatment option for the symptoms caused by neutropenia/neutrophil dysfunction (e.g., mucosal lesions, inflammatory bowel disease) [20].

Several congenital disorders of glycosylation (CDG) have also been associated with neutropenia. Genetic variants in *G6PC3* and *VPS13B* affect the number and function of neutrophils causing different forms of severe congenital neutropenia (SCN). G6PC3-CDG and VPS13B-CDG patients present Dursun and Cohen syndrome, respectively [22]. Neutropenia with progression to bone marrow failure, recurrent infections, combined immunodeficiency, associated with skeletal dysplasia (resembling Desbuquois dysplasia) represent the main clinical presentation of PGM3-CDG [23,24].

### 3.6. Pancytopenia

Hematological abnormalities are common in methylmalonic (MMA), propionic (PA), and isovaleric (IVA) acidemias/acidurias. They can present with acute, chronic, or intermittent symptoms, including failure to thrive, vomiting, neurological deterioration with hypotonia, irritability, and lethargy accompanied by metabolic acidosis, ketosis, hyperlactataemia, hyperammonaemia, and hypoglycaemia [25]. Late-onset patients present with neurological symptoms (encephalopathy, developmental delay), and cardiac and kidney manifestations. Pancytopenia (especially neutropenia) at an initial presentation or during metabolic decompensation as well as during the chronic disease course has been frequently described.

In summary, when faced with megaloblastic anemia, pediatricians should consider intracellular cbl and folate defects while also considering microcytic anemia, iron deficiency and sideroblastic anemia, and porphyrias. Thrombocytopenia with hepatosplenomegaly should prompt an investigation for Gaucher disease and ASMD. Differential diagnosis of neutropenia should include Barth syndrome, GSD Ib, and several CDG.

## 4. Laboratory (Biochemical) Markers of Liver Damage/Function

The liver is an organ where biochemical pathways crucial for the proper functioning of the body occur; thus, many IMDs manifest particularly intensely in the liver [26]. In all liver pathologies, regardless of its etiology, in the initial stages of the disease, liver volume enlargement is most commonly observed. Table 3 presents algorithms useful in the differential diagnosis of IMDs depending on the clinical and biochemical phenotype associated with liver involvement [26].

A spectrum of conditions, ranging from transient and benign to chronic and progressive, may be indicated by elevated serum aminotransferases (transaminases) activity in children. Most IMDs are characterized by hepatocellular damage, manifesting as mild to moderate (3 to 10 times the upper limit of normal) elevations in transaminases activity. An exception is found in diseases associated with the storage of macrophages of the reticuloendothelial system, (lysosomal storage diseases, especially Gaucher disease), where ALT activity is usually normal, but moderate to severe liver and spleen enlargement is a characteristic feature (Table 3) [17]. In both acid sphingomyelinase deficiency (ASMD, formerly Niemann–Pick disease type A, B, A/B) and lysosomal acid lipase deficiency (LAL-D), hepatosplenomegaly with increased activity of transaminases is observed [17].

Cholestasis is not a characteristic feature of IMDs, with some exceptions. Prolonged (cholestatic) jaundice in the newborn (usually accompanied by splenomegaly) may be the first noticeable symptom of Niemann–Pick type C (NPC) disease, preceding the onset of neurological features (Table 3) [27].

In turn, cholestatic jaundice in the newborn, with hepatomegaly or hepatosplenomegaly, accompanied by severe hypotonia, seizures, and characteristic craniofacial features, occurs in Zellweger spectrum disorders (ZSD) [28]. In an early-onset ZSD, the prognosis is poor and survival is usually not beyond the first year of life [28].

Neonatal–infantile cholestasis (usually transient) occurs in the course of inborn errors of bile acid synthesis [29]. 3β-Hydroxy-Δ5-C27-steroid oxidoreductase and Δ4-3-oxosteroid 5β-reductase deficiency are characterized by an isolated liver involvement—they present with infantile cholestasis (which may be accompanied by hepatomegaly and elevated transaminases activity) and malabsorption of fat-soluble vitamins (vitamin D deficiency rickets, coagulopathy, steatorrhea). Infantile cholestasis (most often transient) may also occur in the course of sterol 27-hydroxylase deficiency (cerebrotendinous xanthomatosis) or α-methylacyl-CoA racemase deficiency.

Liver synthetic function impairment, namely coagulopathy (prolonged INR/prothrombine time), is a characteristic feature of some IMDs. Classical galactosemia (deficiency of galactose-1-phosphate uridyltransferase, GALT) tyrosinemia type 1, hereditary fructose intolerance, urea cycle defects, and primary mitochondrial diseases constitute the most common causes of acute liver failure (ALF) among IMDs (Table 3) [26]. Tyrosinemia type 1 most often manifests in the first weeks of life as ALF with moderate hepatocellular damage and cholestasis. In the case of GALT deficiency, symptoms appear in the neonatal period, after the initiation of feeding with foods containing galactose (mother’s milk), as vomiting, diarrhea, cholestatic jaundice, and ALF development. In the case of hereditary fructose intolerance, symptoms appear when fructose is introduced into the diet (most often around 4–6 months of age). In children chronically receiving (some) fructose into the diet, in addition to liver disease—elevated aminotransferase activity, cholestasis, coagulation disorders—kidney damage similar to tubulopathy (Fanconi syndrome) is observed.

Reye’s syndrome or Reye-like syndrome (hepatic encephalopathy with fatty liver) may be the first clinical manifestation of fatty acid oxidation (FAO) defects [30,31,32]. The age of presentation for FAO defects varies widely, with symptoms appearing from the neonatal period through adulthood. The disease may present suddenly as an acute metabolic decompensation, triggered by a prolonged fasting, fever during infection, or increased physical exertion. In addition to signs of liver damage (elevated aminotransferase activity, prolonged INR, and, rarely, cholestasis), myocardial involvement (cardiomyopathy, arrhythmias), myopathy (elevated CK activity, myoglobinuria), and hypoketotic hypoglycemia are usually observed (Table 4) [30,31,32].

Mitochondrial hepatopathies involving respiratory chain defects often present with ALF in the first weeks/months of life. Isolated oxidative phosphorylation (OXPHOS) deficiencies are relatively rare (i.e., isolated complex III deficiency associated with a defect in the *BCS1L* gene—GRACILE syndrome (growth retardation, aminoaciduria, cholestasis, iron overload, lactic acidosis, and early death) or isolated complex IV deficiency associated with a defect in the *SCO1* gene) [33,34]. Combined deficiencies of respiratory chain complexes are more commonly observed (Supplementary Table S1).

The group of primary respiratory chain complex deficiencies includes mtDNA depletions, defined as a reduction in mtDNA copy number, leading to an insufficient synthesis of respiratory chain complexes. These include defects in genes responsible for nucleotide synthesis (e.g., *TK2*, *SUCLA2*, *SUCLG1*, *RRM2B*, *DGUOK*, *MPV17*) or mtDNA replication (*POLG*, *C10orf2*) [35,36]. From a clinical perspective, mtDNA depletion can have the following phenotypes: (i) myopathic (*TK2*, *POLG*, *RRM2B* genes); (ii) encephalomyopathic (*RRM2B*, *SUCLA2*, *SUCLG1* genes); (iii) hepatocerebral (*POLG*, *C10orf2*, *DGUOK*, *MPV17*, *SUCLG1* genes); (iv) neurovisceral (MNGIE—*TYMP*, *RRM2B*, *POLG* genes) [35,36].

Clinical manifestations of hepatocerebral mtDNA depletion syndromes most often include ALF in the first weeks/months of life (Supplementary Table S1). In Alpers–Huttenlocher syndrome, the onset of symptoms may occur later, and liver failure may be induced by valproate, used in the treatment of epilepsy. However, mitochondrial hepatopathies associated with mtDNA depletion syndromes may also present later in life as isolated elevated aminotransferase activity, hepatic steatosis, or cirrhosis. Most patients also present with various neurological symptoms, which may be present from birth or develop concurrently with liver dysfunction, and these sometimes appear only after organ transplantation; DGOUK deficiency may present as isolated liver damage (Supplementary Table S1).

In summary, most IMDs present with elevated transaminases. When co-occurring with neonatal jaundice, Niemann–Pick type C, Zellweger spectrum disorders, and inborn errors of bile acids synthesis should be ruled out. ALF should prompt an investigation for GALT deficiency, tyrosinemia type 1, hereditary fructose intolerance, urea cycle defects, fatty acids oxidation defects, and primary mitochondrial diseases (especially mtDNA depletion syndromes).

## 5. Serum Creatine Kinase

Unlike other muscle enzymes found in skeletal muscle (e.g., lactate dehydrogenase, aldolase, and transaminases), creatine kinase (CK) has relative predominance in the skeletal muscle [37].

Normal serum ranges vary by ethnicity, gender, and age [37]. Detection of elevated serum CK is an indicator of muscle injury, but elevation is not specific to the cause (Supplementary Table S2).

The highest CK levels (over 10 x ULN) are usually observed in dystrophinopathies (Duchenne muscular dystrophy, DMD) and rhabdomyolysis [38,39,40]. In conditions such as spinal muscular atrophy and Guillan–Barre syndrome there may be an elevation of CK, usually no more than five times normal [39].

Metabolic myopathies encompass glycogen storage disorders (GSDs), fatty acid oxidation (FAO) defects, and primary mitochondrial disorders (PMDs) [38,39].

Mixed, hepatic, and muscular (skeletal and cardiac) phenotype is the most (85%) prevalent form of GSD III (Cori–Forbes disease or glycogen debranching enzyme deficiency), namely GSD IIIa. A mild muscle weakness, motor developmental delay (delayed sitting, delayed standing upright, delayed onset of walking), exercise intolerance, and hypotonia are typically presented in the infancy and early childhood of patients with GSD type IIIa. Muscle weakness can be both proximal and distal, and is usually accompanied by hypoglycemia, hepatomegaly, and permanently moderately elevated CK activity in children [41,42].

Patients with GSD V (McArdle disease, muscle glycogen phosphorylase deficiency) usually present in young adulthood with recurrent muscle cramps and pigmenturia with higher-intensity exercise, and most report a second-wind phenomenon (significant reduction in the perception of effort after a few minutes of activity co-temporal with the delivery of blood-borne substrates) [41,42]. Marked CK elevation is a cardinal feature during exercise-induced myalgia and rhabdomyolysis.

Clinical symptoms in GSD VII (Tarui disease, muscle phosphofructokinase deficiency) are very similar to GSD V with exercise-induced pain, muscle cramps, and fatigue; however, they are more severe and often already present during childhood, and can be accompanied by haemolytic anemia and hyperuricaemia [41,42]. Clinically, patients with GSD VII do not show a second-wind phenomenon (Table 5).

Pompe disease (PD), a lysosomal storage disease associated with acid maltase deficiency, is characterized by a highly variable clinical presentation, ranging from asymptomatic elevation in serum CK levels to severe limb muscle weakness and chronic respiratory failure. The greatest CK elevation is usually found in the infantile-onset form of PD (as high as 2000 UI/L) [43]. In the infantile-onset form, severe cardiomyopathy and muscular hypotonia are the cardinal features. Approximately 95% of the late-onset patients have an elevated CK; however, some adults may have CK levels within the normal reference range. In most patients, involvement of the proximal muscles of the lower limbs occurs first, followed by involvement of the proximal muscles of the upper limbs and also chronic respiratory failure [43].

FAO defects are characterized by an episodic course of muscle weakness, rhabdomyolysis, and myoglobinuria (Table 4 and Table 5) [31,32,38,39]. Triggers usually include infections, long duration/vigorous exercise, general anesthesia, fasting, or certain drugs. On the other hand, the association of ichthyosis and myopathy is highly suggestive for Chanarin–Dorfman syndrome.

The systemic form of primary carnitine deficiency is usually characterized by bouts of rhabdomyolysis and hypoketotic hypoglycemia, generalized muscle weakness, and Reye-like syndrome, while serum CK levels can be variable, from normal to 15 times the normal values [38]. The myopathic form of CPT II typically presents with exercise intolerance and recurrent attacks of muscle weakness, muscle cramps, and severe bouts of rhabdomyolysis (CK 50,000–200,000 UI) with myoglobinuria [38].

Primary mitochondrial disorders (PMDs) could be characterized as occurring at any age, with any clinical symptom of varying severity, any constellation of symptoms, and any clinical course (Supplementary Table S3). Frequently, metabolic myopathies in PMDs begin with non-specific symptoms, including myalgia, fatigue, muscle cramps, or exercise intolerance [38,39,44,45,46]. Muscle weakness can first occur in the extraocular eye muscles or the lid elevators, leading to ptosis and external ophthalmoparesis (progressive external ophthalmoplegia, PEO). Apart from limb muscles, axial and respiratory muscles may also be subsequently involved. Some patients may also present with rhabdomyolysis [38,39,44,45,46]. PEO is rarely an isolated finding in children, while myopathy is usually one of the features of multi-system mitochondrial dysfunction (Table 6) [44,45,46].

In summary, IMDs associated with elevated serum CK activity include glycogen storage diseases, fatty acids oxidation defects, and primary mitochondrial disorders. Two clinical forms of metabolic myopathies could be distinguished: those with static or progressive muscle weakness, such as GSD II, III, and IV, and those for which the symptomatology is acute and relapsing, with exercise-induced myalgia with or without rhabdomyolysis, such GSD V and other GSDs, FAO defects, and mitochondrial myopathies.

## 6. Serum Alkaline Phosphatase (ALP)

ALP comprises four different isoenzymes, namely intestinal, placental, germ cell, and tissue non-specific or liver/bone/kidney [47]. In healthy children, the bone and liver fractions of ALP are predominant. Sex and age and also BMI may act as confounding factors for ALP reference ranges; while its activity is highest in the first year of life, it decreases to a plateau, and then rises again during puberty before declining to adult levels [48,49].

### 6.1. Elevated ALP Activity

Differential diagnosis of elevated serum ALP should include the following: (i) bone disorders (rickets, fractures, juvenile Paget’s disease, and bone malignancies); (ii) hepatic diseases (viral hepatitis, cholestasis); (iii) kidney diseases (chronic renal failure, renal tubular acidosis, and other tubulopathies); (iv) drugs (antibiotics, anticonvulsants); (v) pregnancy [50].

Elevated ALP activity (hyperphosphatasia), associated with intellectual disability, and neurological features, mainly seizures, were reported as hyperphosphatasia with mental retardation (HPMRS) syndrome [51].

HPMRS is caused by pathogenic variants in one of the four genes related to biosynthesis of the glycosylophosphatidylinositol (GPI) anchor in the endoplasmic reticulum (*PIGV*, *PIGO*, *PIGW,* and *PIGY*), or two genes related to the post-GPI attachment to proteins (PGAP) type 2 (*PGAP2*) and type 3 (*PGAP3*) [52,53]. The clinical presentation is heterogeneous; however, in addition to the above-mentioned features, gastrointestinal/anorectal abnormalities (Hirschprung’s disease, anterior anus, anal stenosis, anal atresia, mild anal prolapse, inguinal hernia), facial dysmorphism (broad nasal bridge, cleft palate, hypertelorism), and abnormalities of extremities (brachytelephalangyhypoplastic finger and toenails, hypoplastic distal phalange) are frequently observed [54].

Benign transient hyperphosphatasemia (BTH) is a condition of a temporary, markedly increased elevation of serum ALP in children who have no evidence of liver or bone disease. Characteristic features of BTH include the age of presentation as less than 5 years, no evidence of bone or liver disease, elevation in both bone and liver ALP isoenzymes, and a return to normal serum ALP values within 4 months [55,56].

Therefore, in the case of an incidental finding of high serum ALP in an otherwise healthy infant or child, it is recommended to repeat the ALP level check within a few months [55,56].

### 6.2. Low ALP Activity

The main diagnostic clue for hypophosphatasia (HPP) is the low (for age and sex) ALP activity. At least two different ALP activity measurements are recommended in patients with suspected HPP.

HPP is caused by pathogenic variants in the *ALPL* gene encoding a tissue non-specific alkaline phosphatase (TNSALP). The clinical presentation is heterogeneous and can include premature teeth loss, rickets, osteomalacia, hypercalcemia, and also pyridoxine-dependent seizures; see Table 7 [57,58,59]. A low ALP level requires the exclusion of other conditions or drugs which can contribute to reduction in its activity [19]; see Supplementary Table S4.

In summary, when faced with elevated serum ALP activity associated with various neurological manifestations, pediatricians should consider hyperphosphatasia with mental retardation syndromes, while low ALP activity combined with skeletal, dental, muscular, or neurological presentations should prompt an investigation for hypophosphatasia.

## 7. Serum Uric Acid

Uric acid, produced mostly in the liver, is the end product of dietary and endogenous purine metabolism (Supplementary Figure S1) and approximately 75% of daily urate is excreted by the kidney. Key enzymes that cause abnormal uric acid levels include phosphoribosyl pyrophosphate (PRPP) synthetase, purine nucleoside phosphorylase (converting inosine to hypoxanthine), xanthine oxidase (converting hypoxanthine to xanthine and subsequently xanthine to uric acid), and hypoxanthin-guanine phosphoribosyltransferase (HGPRT; reconverting hypoxanthine and guanine into their respective nucleotides) [60].

### 7.1. Hyperuricaemia

Serum urate concentrations in most children range from 3 to 4 mg/dL. Hyperuricemia should be considered when the serum uric acid concentration is ≥6 mg/dL in girls, ≥6 mg/dL in boys younger than 15 years of age, and ≥7 mg/dL in boys older than 15 years [61]. It could be related to UA overproduction, decreased renal UA excretion, or a combination of both mechanisms (Supplementary Table S5). A quantitative determination of UA in urine, as the UA/creatinine ratio, is thus mandatory to obtain.

Primary hyperuricaemia is related to UA overproduction occurring in Lesch–Nyhan disease (LND), characterized by a severe deficiency of HPRT, resulting in accumulation of its substrates, namely guanine and hypoxanthine [62,63]. Despite this overproduction, marked increase in the serum uric acid level is prevented by efficient renal clearance. Psychomotor delay is usually the first clinical sign to become apparent within 3 to 6 months of age. During the first year of life, serum and urine uric acid determinations should be included in the differential diagnosis of psychomotor delay [63]. Severe generalized dystonia, superimposed on a baseline hypotonia, may lead to an inability to stand up and walk, and patients must use a wheelchair. Other neurological features include involuntary movements (choreoathetosis, ballismus), dysarthria and dysphagia and opisthotonus, and severe intellectual disability. Compulsive self-injurious behavior is the most striking feature of LND. In the same year (1967) of describing LND, Kelly, Greene, Rosenbloom, Henderson, and Seegmiller reported a partial deficiency of HPRT activity associated with gout and no neurological involvement (termed as Kelley–Seegmiller syndrome) [63].

Other IMD resulting in hyperuricemia are related to UA overproduction due to nucleotide depletion (increased ATP consumption or impaired ATP regeneration) and include the following: (i) hereditary fructose intolerance; (ii) fructose-1,6-biphosphatase deficiency; (iii) GSD types III, V, and VII; (iv) MCAD (medium-chain acyl-CoA dehydrogenase) deficiency [62].

Regarding GSD Ia, hyperuricaemia results from excessive UA production combined with an impaired UA renal excretion (hypouricosuria) [62].

### 7.2. Hypouricaemia

Hypouricemia is usually defined by a serum uric acid level of <2.0 mg/dL. Differential diagnosis should include primary or secondary tubulopathy, decreased production, or increased urinary excretion (Supplementary Table S6) [61].

Decreased UA production comprises purine nucleoside phosphorylase (PNP) deficiency and xanthinurias I, II, and III (molybdenum cofactor deficiency) [62].

In PNP deficiency very low UA levels in plasma and urine are caused by the block in guanine and hypoxanthine formation [64]. PNP deficiency causes a rare combined immunodeficiency disorder with profound T cell deficiency (with variable B and NK cell functions) resulting in recurrent and persistent infections typically beginning in infancy or early childhood. Neurologic features, including ataxia, hyper/hypotonia, developmental delay, muscle stiffness (spasticity), are also observed. It should be noted that not only normal but also low levels of serum uric acid could be observed in PNP deficiency [64].

Xanthine oxidoreductase (XOR) catalyzes two hydroxylation steps in the metabolic pathway of purine degradation, namely hypoxanthine to xanthine and xanthine to uric acid. XOR has two forms: xanthine dehydrogenase (XDH) and xanthine oxidase (XO). XOR deficiency is classified into three types. Type I is caused by xanthine dehydrogenase/oxidase deficiency (XDH/XO), and type II results from a combined deficiency of XDH/XO and aldehyde oxidase (AOX) caused by dysfunctional variants in the molybdenum cofactor sulfurase (MOCOS), which is required for XDH/XO and AOX activity [65]. The major clinical manifestation of xanthinurias type I and II is urolithiasis which can present as hematuria, crystalluria, recurrent urinary tract infections, renal colic, and acute and chronic renal failure. Less frequent manifestations are myopathy, arthropathy, and duodenal ulcers.

The third type of xanthinuria is caused by defects in molybdenum cofactor (MoCo) synthesis leading to MoCo deficiency (MoCD) [66]. The early-onset form of MoCD (caused by MOCS1 deficiency, also called MoCD-A) typically presents as neonatal encephalopathy with intractable seizures, brain atrophy, lens dislocation, and a high mortality [66].

### 7.3. Normouricaemia and Normouricosuria

This category comprises the purine IEM Adenosine deaminase (ADA) deficiency, adenine phosphoribosyltransferase (APRT) deficiency, and adenylosuccinate lyase (ADSL) deficiency [62].

ADA deficiency is a severe combined immunodeficiency (SCID), otherwise known as ADA-SCID [67]. In addition to immunological features, skeletal abnormalities (costochondral abnormalities, skeletal dysplasia), cerebral (cognitive and behavioral defects), and hepatic and pulmonary features are observed.

Most of the patients with adenylosuccinate lyase (ADSL) deficiency present with a severe form (type I) characterized by severe psychomotor retardation, early onset of seizures, and microcephaly [68]. Patients with type II (moderate or mild form), who develop symptoms within the first years of life, usually suffer from slight to moderate psychomotor retardation and transient contact disturbances, while seizures, if present, appear between the second and fourth year of life.

In summary, differential diagnosis of hyperuricemia should include Lesch–Nyhan disease (psychomotor delay, severe dystonia), ketotic GSD types (III, VI, IX), hereditary fructose intolerance, and fructose-1,6-biphosphate deficiency. When faced with hypouricemia, pediatricians should consider purine nucleoside phosphorylase (PNP) deficiency and xanthinurias I, II, and III.

## 8. Hypoglycaemia

There is still no clear definition of hypoglycemia in children. According to the American Academy of Pediatrics (AAP) and Pediatric Endocrine Society (PES), hypoglycemia is diagnosed as following: (i) plasma glucose level < 47 mg/dL in at-term newborns during first 48 h of life; (ii) plasma glucose level < 50 mg/dL in at-term newborns after 48 h of life, infants, and younger children; (iii) Whipple’s triad characterized by signs and/or symptoms of hypoglycemia, reduced plasma glucose concentration, and resolution of these signs/symptoms after acquisition of normoglycemic status in older children [69,70].

**Figure 1:**
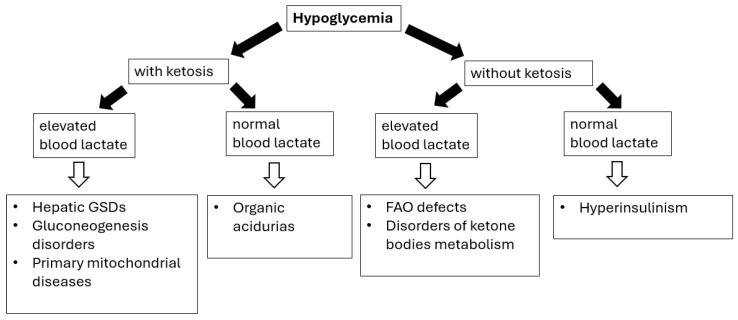
Hypoglycemia in IMDs—diagnostic algorithm. Black arrows provide the next diagnostics (biochemical) steps while white arrows with black outlines provide the final diagnoses of certain IMDs.

The main causes of hypoglycemia can be classified into endocrine (including hyperinsulinism, growth hormone deficiency, cortisol deficiency, and increased Insulin-Like Growth Factor-II secretion during Wilms’ tumor, nephroblastoma, dysgerminomas, and lymphomas/leukemias) or metabolic etiology (Figure 1) [69,70].

Diagnosing hypoglycemia requires the following: (i) detailed physical examination (especially regarding liver size/volume) as the presence of hepatomegaly (see Table 3) can help differentiating disorders causing fasting ketotic hypoglycemia; (ii) performing a blood glucose profile, with particular emphasis on determining blood glucose levels after the longest periods of breastfeeding (after a night-time break) or as needed if alarming symptoms occur; (iii) laboratory investigations (correlation with other metabolic features), including the presence (or absence) of lactic acidosis and ketonuria/ketonaemia [69,70,71].

The timing of hypoglycemia is the crucial issue; the patients’ fasting tolerance can provide an essential clue to the diagnosis in children with fasting hypoglycemia (hypoglycemia after a short fast suggests hepatic GSD, while after a moderate to long fast suggests gluconeogenesis defects or FAO/ketone bodies defects) [69,70,71].

### 8.1. Glycogen Storage Disorders

A characteristic feature of hepatic glycogen storage diseases (GSDs), including GSD I, III, IV, VI, IX, and XI, is hepatomegaly and hypoglycemia [71]. In GSD type IV, unlike other forms of GSD, hypoglycemia (and ketosis) is not observed until end-stage liver failure [72].

Patients with GSD I may experience severe hypoglycemia (impaired glycogenolysis and gluconeogenesis) as early as infancy (usually 3–6 months of age)—usually several hours after a meal, accompanied by a severe lactic acidosis [20,21,71]. Other characteristic features of GSD type I include a short stature, hepatomegaly (in most cases also nephromegaly), hyperlipidemia (including hypertriglyceridemia due to increased triglyceride production as an indirect consequence of impaired gluconeogenesis), and hyperuricemia (increased production and decreased excretion of uric acid) [20,21,71]. Kidney damage progresses over time and manifests itself through damage to both the renal tubules (tubulopathy) and the glomeruli (hyperfiltration mechanism, hypertension). Hypercalciuria and hypocitraturia increase the risk of nephrocalcinosis and urolithiasis. Patients with GSD Ib also exhibit neutropenia and impaired neutrophil function, which predispose them to recurrent bacterial and fungal infections, and oral ulcers [20]. Patients are also at risk of developing inflammatory bowel disease (Crohn’s-like). In the prolonged fasting test in GSD III/VI/IX, especially in type IX, hypoglycemia may not occur even after several hours of fasting [71,72,73]. In patients with GSD III/VI/IX, hypoglycemia (usually after prolonged fasting or during illness) is not as severe as in GSD I due to normal gluconeogenesis. However, significant ketosis is a characteristic feature. The degree of hepatomegaly is similar in GSD III, VI, and IX [71,72,73,74]. The severity of hypoglycemia, increased aminotransferase activity, and hyperlipidemia are usually more severe in GSD III than in GSD VI and IX. Hepatomegaly and elevated aminotransferase activity in most patients with GSD III/VI/IX resolve with age—the apparent improvement may be related to a reduced relative glucose requirement [71,72,73,74].

GSD type XI (Fanconi–Bickel syndrome) is caused by a defective glucose–galactose transporter (GLUT2) found in hepatocytes, pancreatic β-cells, enterocytes, and renal tubular cells [71]. Symptoms typically begin between 3 and 10 months of age and include hepatomegaly, failure to thrive, fasting ketotic hypoglycemia, and postprandial hyperglycemia. Proximal tubular dysfunction leads to glucosuria, hypercalciuria, hyperphosphaturia, hypophosphatemia, aminoaciduria, albuminuria, and hyperchloremic acidosis with a normal anion gap. In older children, short stature, hypophosphatemic rickets, osteopenia, and delayed puberty are observed.

### 8.2. Gluconeogenesis Disorders

Deficiency of fructose-1,6-bisphosphatase (FBP), a key enzyme of gluconeogenesis, usually occurs in the first year of life through ketotic hypoglycemia and lactic acidosis triggered by catabolic episodes such as prolonged fasting or febrile infections. Mild hepatomegaly, elevated aminotransferases activity, and sometimes acute liver failure are also observed [69,70].

Pyruvate carboxylase deficiency is a defect involving both gluconeogenesis and the Krebs cycle, usually presenting with neurological manifestation (developmental delay, epilepsy, encephalopathy), fasting hypoglycemia, lactic acidosis, hyperammonemia, and sometimes hypoglycemia [69,70].

Glycerol kinase deficiency (GKD) can present either isolated or together with congenital adrenal hypoplasia or Duchenne muscular dystrophy (partial deletion of Xp21). Patients with isolated GKD can develop episodic vomiting with hypoglycemia, ketosis, metabolic acidosis, and coma [69,70].

### 8.3. FAO Defects and Disorders of Ketone Body Metabolism

Similarly to FAO defects (see Section 5), prolonged (>8 to 24 h) fasting and intercurrent illness are triggers for metabolic decompensation. Ketogenesis defects are characterized by hypoketotic hypoglycemia with or without hyperammonemia, metabolic acidosis, and liver disease [69,70]. Decompensation can lead to encephalopathy, vomiting, and coma. Conversely, ketolysis defects present with episodes of hyperketotic hypoglycemia and severe ketoacidosis in childhood [69,70].

### 8.4. Idiopathic Ketotic Hypoglycemia

Idiopathic ketotic hypoglycemia (IKH) is one of the most frequent forms of hypoglycemia in healthy children aged 18 months to 5–7 years [69,70]. Typically, the child presents with symptomatic hypoglycemia after a long fast often precipitated by an intercurrent illness. Biochemical abnormalities include the presence of hypoglycemia with massive ketosis and metabolic acidosis. The child improves dramatically on dextrose infusion (conversely glucagon injection elicits little or no increase in glucose concentrations) and is usually restored to normal health within hours.

In summary, when faced with hypoglycemia, correlation with other metabolic features, including the presence (or absence) of lactic acidosis and ketonuria/ketonaemia, is mandatory. IMDs associated with hypoglycemia include hepatic glycogen storage diseases (types I, III, IV, VI, IX, and XI), gluconeogenesis disorders (ketotic hypoglycemia), and defects of fatty acids oxidation and ketone bodies metabolism (hypoketotic hypoglycemia).

## 9. Hyperlactatemia with or Without Lactic Acidosis

The definition of elevated lactate levels is not universally standardized. However, in healthy children, normal venous lactate levels typically range from 0.5 to 2.2 mmol/L [75]. In clinical practice, it is important to distinguish between hyperlactataemia and lactic acidosis, as they constitute two distinct clinical conditions. While hyperlactataemia is characterized by a moderate increase in blood lactate levels (ranging from 2 to 4 mmol/L) without concomitant metabolic acidosis, lactic acidosis is characterized by significantly elevated lactate levels, usually exceeding 5 mmol/L, accompanied by metabolic acidosis (pH < 7.35, bicarbonate < 18 mmol/L) [75].

**Figure 2:**
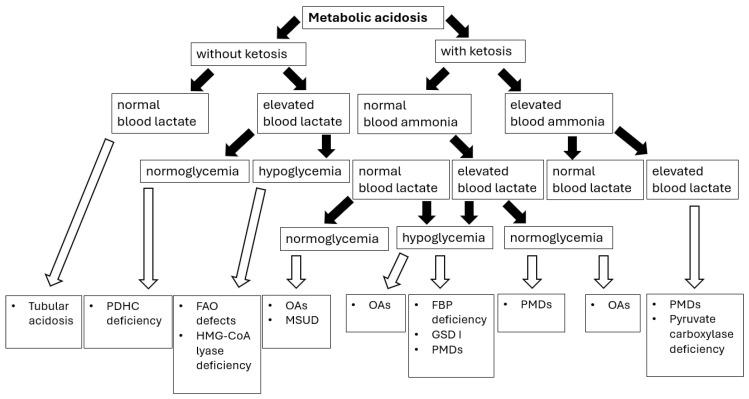
Metabolic acidosis in congenital metabolic diseases—diagnostic algorithm [76]. Abbreviations: PDHC—pyruvate dehydrogenase complex; FAO—fatty acids oxidation; OAs—organic acidurias; GSD I—glycogen storage disease type I; PMDs—primary mitochondrial diseases; FBP—fructose-1,6-biphosphate. Black arrows provide the next diagnostics (biochemical) steps while white arrows with black outlines provide the final diagnoses of certain IMDs.

Sampling blood for blood gas analysis and ionogram in a patient with metabolic acidosis allows for the calculation of the anion gap LA = [Na^+^] − ([Cl^−^] + [HCO_3_^−^]), thus enabling us to establish a preliminary etiology of metabolic acidosis (Figure 2).

The normal LA value in children < 3 years of age is in the range of 10–14 mEq/L, and in older children 10–18 mEq/L [76]. In many IMDs presenting with lactic acidosis, an increased anion gap (>16) is observed due to the accumulation of solid acids such as lactic acid, ketoacids, and other organic acids. A normal anion gap (hyperchloremic acidosis) may indicate a renal cause of metabolic acidosis [76].

In 1976, Cohen and Woods proposed a classification of hyperlactataemia into two types: type A, which was associated with inadequate tissue oxygen delivery, and type B, which occurred in the absence of tissue hypoxia. Most clinical cases of lactic acidosis, both in children and adults, are not due to hypoxia [75].

The other classification of hyperlactatemia, based on the pathophysiology of lactate accumulation, was recently proposed by Muller et al. [77]. Elevated serum lactate levels can occur under three main conditions: (i) increased pyruvate production, including non-specific glycolysis stimulation (e.g., respiratory alkalosis), malignancy-associated metabolic disturbances, and other causes influencing the redox potential of the cell (e.g., diabetic ketoacidosis); (ii) decreased pyruvate utilization, observed in hypoxia-related hyperlactataemia, pyruvate dehydrogenase dysfunction (thiamine deficiency and inherited enzymatic dysfunction), and mitochondrial dysfunction (PMDs, drugs); (iii) decreased lactate clearance observed in liver and renal failure [77].

Lactic acidosis is the most well-known laboratory finding in patients with PMDs. Low ATP levels activate glycolysis, which overproduces pyruvate. Extra pyruvate is either converted to lactate or transaminated to alanine. The lactate-to-pyruvate ratio is a well-established indicator of the cytoplasmic NADH/NAD^+^ redox ratio and is clinically advantageous in that it can differentiate disorders of pyruvate metabolism like pyruvate dehydrogenase complex (PDHC) deficiency, in which patients would classically have proportional elevations of both biomarkers and therefore a normal ratio (less than 20), as opposed to other PMDs in which lactate is comparatively higher than pyruvate [78]. PDHC deficiency comprises one of the most common congenital lactic acidosis (cytoplasmic reduction to lactate or transamination to alanine) with the majority of cases resulting from a defect in the E1alpha subunit (X-linked, thiamine-dependent form) [79,80]. It should be considered in every case of neurological disorders with lactic acidosis and lactate-to-pyruvate ratio less than 20. PDHC deficiency could manifest as an early-onset encephalopathy with microcephaly and brain malformations (ventricular dilatation, corpus callosum dysgenesis, Leigh syndrome). However, the neurological presentation could be heterogeneous, including psychomotor delay, axial hypotonia, recurrent encephalopathy with seizures, axonal neuropathy, progressively worsening movement disorders, and spasticity [79,80].

Clinically, elevated blood lactate levels are observed in various IMDs, not only limited to PMDs (Table 8). Most IMDs presenting with hyperlactataemia are accompanied by ketosis, with the exception of pyruvate dehydrogenase deficiency, GSD I, or certain FAO defects. Most FAO defects (see Table 4) that present with hypoketosis are sometimes accompanied by elevated plasma lactic acid during decompensation. Hypoglycemia is a major stimulus for glycogenolysis and gluconeogenesis; therefore, enzyme deficiencies which impair gluconeogenesis (Table 8) cause increased pyruvate production. In gluconeogenic defects, such as fructose-1,6-biphosphatase deficiency and GSD Ia, hyperlactataemia peaks during fasting or hypoglycemia. In disorders affecting glycogen degradation (see Section 8.1), hyperlactataemia peaks postprandial. In hepatic glycogen synthetase deficiency (GSD 0), instead of the other defects in glycogen degradation, there is no liver enlargement present as there is an absence of glycogen formation/accumulation in the liver [81]. In pyruvate carboxylase deficiency, hyperlactataemia occurs in both the fasting and fed states.

In summary, lactic acidosis in IMDs is usually associated with an increased anion gap. Besides pyruvate dehydrogenase complex deficiency, primary mitochondrial disorders (PMDs) are associated with both elevated lactate and pyruvate, and thus an increased lactate-to-pyruvate ratio. When faced with lactis acidosis, pediatricians should rule out also defects of biotin metabolism, fatty acids oxidation, organic acids metabolism, and carbohydrate metabolism (gluconeogenesis disorders, glycogen storage diseases, hereditary fructose intolerance).

## 10. Hyperammonemia

Ammonia should always be measured in the following cases: (i) unexplained altered level of consciousness; (ii) neurological disorder of an unknown cause; (iii) liver failure of unknown cause; (iv) intoxication syndrome [82,83].

In healthy individuals, and with proper collection, the ammonia concentration should not exceed 50 μmol/L (80 μg/dL), with the exception of the neonatal period (<110 μmol/L (180 μg/dL)) [82,83]. To prevent false results secondary to hemolysis, blood must be collected with the use of an anticoagulant (EDTA or heparin) and preferably in a chilled tube (Table 9).

A defect in one of the six urea cycle enzymes and two membrane transporters results in so-called primary hyperammonemia (urea cycle disorders, UCDs), while metabolic defects outside the urea cycle as well as side effects of drugs can lead to secondary hyperammonemia (Table 9) [82,83]. Hyperammonemia is the hallmark of UCDs with peak ammonia concentrations > 500 μmol/L in most neonatal patients at presentation [82,83]. In the context of acute neonatal encephalopathy, severe hyperammonemia (>500 μmol/L) is generally caused either by a UCD (with respiratory alkalosis and no ketosis) or by organic acidurias (MMA, PA with metabolic acidosis, ketosis). Normal ammonia virtually excludes a UCD in a symptomatic newborn (but not in an older patient).

Clinically, UCDs can manifest for the first time at any age, but approximately two-thirds of cases are observed in the neonatal period (early manifestation). Patients with complete enzyme deficiency usually develop acute hyperammonemic encephalopathy in the first days of life (Table 10) [82,83]. However, the symptoms could be non-specific (Table 10) and resemble sepsis-like or intoxication syndromes in the course of other IMDs. It should be noted that hypoglycemia, acidosis (respiratory alkalosis), or significant ketosis are not observed (this does not apply to the rare form—carbonic anhydrase Va deficiency) [82,83].

Hyperammonemia is always an emergency situation. Immediate ammonia measurement in an emergency setting is crucial since patient outcome correlates both with the length and peak value of hyperammonemia. Treatment should start immediately after elevated plasma ammonia has been found and before any specific diagnosis is made. In any patient with hyperammonemia, amino acid profiles are necessary not only for differential diagnosis in newly diagnosed patients but also for optimal treatment.

In summary, hyperammonemia needs simultaneous differential diagnosis and treatment. When facing hyperammonemia, pediatricians should consider urea cycle defects, organic acidurias, and fatty acids oxidation defects.

## 11. Lipid Profile

Cholesterol levels fluctuate with age and developmental stages [84,85,86,87]. The classification of total, LDL, and HDL cholesterol was published in the Report of the National Education Cholesterol Program (NCEP)—Expert Panel on Blood Cholesterol Levels in Children and Adolescents [84,85,86].

### 11.1. Hypercholesterolemia

Familial hypercholesterolemia (FH) is the most common monogenic disease, caused by pathogenic variants in one of the following genes: *LDLR*, *PCSK9*, *APOB*, *APOE*, *LDLRAP1* [86]. It leads to an accelerated development of atherosclerotic cardiovascular disease. The homozygous form (HoFH) results in highly elevated LDL-C levels (>500 mg/dL), while a heterozygous form (HeFH) is much more common; however, it is typically asymptomatic in children and adolescents. Current guidelines emphasize the importance of initiating lipid-lowering therapy early in children with FH; thus, an early diagnosis at 5 years of age is recommended [86].

Several factors can elevate total serum cholesterol (TC), LDL-C, and triglycerides (TG) levels, occasionally leading to concentrations resembling those observed in FH. Causes of secondary hypercholesterolemia include the following: (i) endocrine disorders (uncontrolled type 1 and 2 diabetes mellitus, metabolic syndrome, hypothyroidism, hepercortisolism); (ii) medications (steroids, oral estrogen, second generation antipsychotics, antidepressants, beta-blockers, sirolimus, antiretroviral therapy, thiazide diuretics); (iii) renal disease (renal failure, nephrotic syndrome); (iv) liver disease (acute hepatitis); (v) excessive alcohol intake; (vi) pregnancy; (vii) chronic inflammatory conditions (systemic lupus erythematosus) [86,87].

Very high triglyceride (TG) levels > 500 mg/dL should prompt diagnostics into lipoprotein lipase (LPL) deficiency (termed as familial chylomicronemia syndrome) or its activator, apoprotein C-II deficiency; see Table 11 [87].

### 11.2. Hypocholesterolemia

Primary hypocholesterolemia is characterized by TC and/or LDL-C levels below the fifth percentile of the general population, adjusted for age and sex—the cut-off value should be set at 3.1 mmol/L (119.9 mg/dL) [88,89]. Several genetic causes of hypocholesterolemia are known, with the most common being familial hypobetalipoproteinemia and abetalipoproteinemia (Table 11). Additionally, hypocholesterolemia could be associated with other genetic conditions, including Smith–Lemli–Opitz syndrome (SLOS) or selected N-glycosylation disorders.

SLOS, caused by a deficiency in 7-dehydrocholesterol reductase, is the most common and best characterized disorder of the cholesterol biosynthesis pathway. The diagnosis is established based on suggestive clinical features (microcephaly, anteverted nares, ptosis, syndactyly of the second and third toes, hypospadias, cryptorchidism, micropenis, psychomotor delay, hypotonia, and feeding disorders) and elevated 7-dehydrocholesterol levels. Although serum cholesterol is usually low, it may be in the normal range in approximately 10% of patients.

In summary, when facing hypercholesterolemia, pediatricians should consider familial hypercholesterolemia as the most frequent disease, while hypocholesterolemia should prompt diagnostics especially into familial hypo-/abetalipoproteinemia (associated with neurological manifestation).

**Table 11:** Selected genetically determined disorders of lipoprotein metabolism. Abrreviations: ↑—elevated.

Disease	Lipoprotein Abnormalities	Characteristic Features
Familial lipoprotein lipase or its activator (apoC-II) deficiency	↑ chylomicrons. ↑ triacylglycerols. N/↑ cholesterol.	Creamy appearance of blood. Enlargement of the liver and spleen (triacylglycerol uptake by macrophages). Recurrent pancreatitis. Xanthelasma (triacylglycerols in skin macrophages).
Congenital deficiency of apoprotein A-I and apoprotein A-II (Tangier disease)	Abnormal chylomicrons and VLDL. Reduced plasma cholesterol concentration.	Enlarged palatine tonsils with an orange tint. Enlarged spleen, liver, and lymph nodes (accumulation of cholesterol esters). Neurological symptoms—sensory disturbances, muscle weakness.
Abetalipoproteinemia (lack of apoprotein B-48, apo-B48)	Chylomicrons, LDL, and VLDL are absent in plasma. Triacylglycerol and cholesterol concentrations are many times lower than physiological values.	Malabsorption syndrome (numerous fat droplets appear in the cytoplasm of intestinal epithelial cells)—steatorrhea, ADEK vitamin deficiency. Neurological disorders—spinocerebellar ataxia, peripheral neuropathy, myopathy, balance disorders, muscle weakness, and spastic muscle contractions. Visual disturbances—visual field defects, twilight vision, and symptoms of retinitis pigmentosa.
Lecithin–cholesterol acyltransferase deficiency	Very low HDL-C. Mild to severe hyperTG. Presence of lipoprotein X (Lp-X). Decreased apo A-I, A-II.	Corneal opacities. Hemolytic anemia. Proteinuria. Renal failure.

## 12. Red Flags—Summary

There is no single clinical or biochemical feature that identifies a specific IMD. However, correlating the occurrence of certain clinical and biochemical features may contribute to a more accurate and timely diagnosis of IMD; see Table 12.

## Figures and Tables

**Table 1:** Usefulness of basic laboratory tests in diagnosis of IMD. Abbreviations: “+++’’ present in >90% of cases; “++’’ present in 60–90% of cases; “+’’ present in 30–60% of cases; “±’’ present in 10–30% of cases; “−‘’ present in <10% of cases. Gray areas—the most characteristic biochemical features/symptoms. Note—not all the symptoms listed may occur simultaneously, as the disease is a dynamic process and the symptoms increase as the disease progresses or decrease with treatment, and some even regress.

	Urea Cycle Disorders	Organic Acidurias	Aminoacidopathies	Inborn Errors of Carbohydrate Metabolism	Fatty Acid Oxidation Defects	Congenital Lactic Acidoses
Metabolic acidosis	Respiratory alkalosis	++	±	±	±	±
Hyperammonemia	+++	++	±	−	±	−
Hypoglycemia	−	±	±	++	++	±
Ketonuria/ketonaemia	−	++	+	+	−	+
Elevated serum lactate	−	±	±	+	±	+++

**Table 2:** Selected biochemical parameters in megaloblastic anemias due to vitamin B12 or folate deficiency [8,9]. Abbreviations: MMA—methylmalonic acid; Hcy—homocysteine; N—normal; ↑—elevated; ↓—decreased; “+’’ present; “+/−‘’ may be present.

	Macrocytosis or Macrocytic Anemia	Total Vitamin B12	Folate	MMA	Hcy
Neonatal cbl deficiency	+	↓	N	↑	↑
Nutritional cbl deficiency	+	↓	N	↑	↑
cblC	+/−	N	N	↑	↑
cblD	+	N	N	N/↑	↑
cblF/cblJ	+	N	N	↑	↑
cblE/G	+	N	N	N	N
Transcobalamin II deficiency	+	N	N	↑	↑
Folate deficiency or malabsorption	+	N	↓	N	↑
DHFR deficiency	+	N	↓	N	N
MTHFD1 deficiency	+	N	↓	N	↑

**Table 3:** Inherited metabolic liver diseases—diagnostic algorithm (* in these diseases, hypoglycemia is associated with glucose or fructose consumption. A common feature is progressive liver and kidney damage in the form of renal tubular acidosis and Fanconi–De Toni–Debree syndrome (hypophosphatemic rickets, glycosuria, aminoaciduria) [26].

Hepatomegaly or hepatosplenomegaly with cholestasis	α-1-antitrypsin deficiency, progressive familial intrahepatic cholestasis, bile acid synthesis disorders, Niemann–Pick type C disease, citrin deficiency, Zellweger syndrome spectrum disorders, adenosine kinase deficiency; less frequently—galactosemia, fructosemia, tyrosinemia type I, congenital disorders of glycosylation
Enlarged liver without evidence of hepatocellular damage with enlarged spleen	Lysosomal storage diseases (e.g., Gaucher disease, mucopolysaccharidoses)
Enlarged liver with signs of hepatocellular damage and possible enlargement of the spleen	Acid sphingomyelinase deficiency (formerly Niemann–Pick disease type A, B, A/B), lysosomal lipase deficiency—early-onset form (Wolman disease) and late-onset form (cholesteryl ester storage disease)
Hepatomegaly with tubulopathy	Galactosemia associated with GALT deficiency, hereditary fructose intolerance, tyrosinemia type I, transaldolase deficiency (TALDO), GSD type XI, congenital disorders of glycosylation (e.g., PMM2-CDG)
Hepatomegaly with hypoglycemia	Hepatic glycogenoses (GSD types I, III, VI, IX, XI), gluconeogenesis disorders (e.g., fructose-1,6-bisphosphatase deficiency), galactosemia associated with GALT deficiency *, hereditary fructose intolerance *, mitochondrial hepatopathies, congenital disorders of glycosylation (e.g., PMM2-CDG, MPI-CDG)
Hepatomegaly with hyperlipidemia	Visceral form of acid sphingomyelinase deficiency (formerly Niemann–Pick disease type B), late-onset form of lysosomal lipase deficiency (cholesteryl ester storage disease), hepatic glycogenoses (GSD I, III, VI, IX), primary defects of lipoprotein metabolism (apo-CII deficiency, lipoprotein lipase deficiency)
Acute liver failure < 3 months of age	Gestational alloimmune liver disease (GALD), galactosemia associated with GALT deficiency, tyrosinemia type I, mitochondrial hepatopathies, urea cycle disorders, fatty acid β-oxidation disorders, Wolman disease, transaldolase deficiency (TALDO), congenital disorders of glycosylation
Acute liver failure at age 3 months–2 years	Galactosemia associated with GALT deficiency, hereditary fructose intolerance, tyrosinemia type I, mitochondrial hepatopathies, urea cycle disorders, fatty acid β-oxidation disorders, transaldolase deficiency (TALDO), congenital disorders of glycosylation
Acute liver failure > 2 years of age	Wilson’s disease, mitochondrial hepatopathies, urea cycle disorders, fatty acid β-oxidation disorders, congenital glycosylation disorders

**Table 4:** Clinical manifestation of FAO defects [30,31,32].

Carnitine transporter (OCTN2) deficiency—primary carnitine deficiency	Early (first 2 years of life) hepato-muscular manifestation with hepatomegaly, elevated transaminases, hypoketotic hypoglycemia, and hepatic encephalopathy; in older children, cardiomyopathy, skeletal muscle weakness, and slightly elevated CK levels.
	Muscular form in an early childhood with dilated cardiomyopathy, hypotonia, muscle weakness, and elevated CK levels.
	Adult form with cardiac arrhythmia in pregnant women, muscle fatigue.
Carnitine-palmitoyl transferase type 1A (CPT1A) deficiency—hepatic form	Isolated liver involvement with hepatomegaly, elevated transaminases, hepatic encephalopathy.
Carnitine-palmitoyl transferase type 2 (CPT2) deficiency	Neonatal/infantile form with severe hepato-muscular manifestation—liver failure, cardiomyopathy, respiratory failure, and/or cardiac arrhythmias.
	Severe childhood hepato-muscular form with liver failure, cardiomyopathy, cardiac arrhythmias, and myopathy.
	Classic muscular form—from infancy to adulthood (onset is generally observed in childhood or early adulthood); recurrent episodes of muscle pain and weakness and rhabdomyolysis; no signs/symptoms of myopathy are observed between attacks.
Very long chain acyl-CoA dehydrogenase deficiency (VLCADD)	Severe infantile form with hypertrophic cardiomyopathy and acute liver failure.
	Hepato-muscular form with a milder course in childhood.
	Adult form with recurrent episodes of rhabdomyolysis.
Long-chain fatty acid 3-OH-acyl-CoA dehydrogenase deficiency (LCHADD)	Newborns with the severe phenotype present with hypoglycemia, hepatomegaly, encephalopathy, and often cardiomyopathy within a few days of birth.
	Intermediate phenotype—hypoketotic hypoglycemia caused by infection or starvation in infancy.
	Mild (late-onset) phenotype—myopathy and/or neuropathy.
	Peripheral neuropathy in adolescence or adulthood (in approximately 80% of patients with MTP deficiency and in approximately 5–10% of patients with LCHAD deficiency)—a slow, progressive sensorimotor polyneuropathy, along with limb–girdle myopathy with recurrent episodes of myoglobinuria. Retinitis pigmentosa—affects approximately 30–50% of patients with LCHAD deficiency and approximately 5–13% of patients with MTP deficiency; deterioration of color vision, vision in low light, and vision in the center of the visual field, up to complete vision loss.
Medium-chain acyl-CoA dehydrogenase deficiency (MCADD)	Before the era of screening, the presentation was similar to CPT1A deficiency: liver failure with hepatic encephalopathy.
	Currently, MCAD deficiency is usually diagnosed before decompensation occurs, treatment is initiated early, and acute metabolic decompensation is rare.

**Table 5:** Clinical manifestation of selected metabolic myopathies [38]. Abbreviations: “+’’ present; “+/−‘’ may be observed.

Disease	Pompe Dis-ease	GSD IIIa	GSD IV	GSD V	GSD VII	GSD X	GSD XI	GSD XIII	GSD XIV	GSD XV	PCD	CPT II	MELAS	MERRF	TK2 Deficiency	Kearn–Sayre Syndrome
Myalgia	+	+		+	+							+	+	+		
Proximal muscle weakness	+	+	+							+	+		+	+	+	+
Distal muscles weakness		+														
Rhabdomyolysis	+/−	+/−		+	+	+	+	+	+			+	+/−	+/−	+	
Second-wind phenomenon				+												
Exercise intolerance	+	+	+	+	+	+	+	+	+	+	+	+	+	+	+	+
Hepatomegaly	+/−	+	+				+		+		+					
Cardiomyopathy	+/−	+	+/−						+		+		+/−			

**Table 6:** Clinical, biochemical, and molecular phenotypes of selected primary mitochondrial disorders with myopathy [44,45,46].

MELAS Mitochondrial encephalomyopathy, lactic acidosis, stroke-like episodes	Onset of symptoms 2–10 years of age: re-current headaches, recurrent vomiting, migraines, seizures Encephalomyopathy with stroke-like episodes (most often with amblyopia) in a location inconsistent with the anatomical course of the cerebral vessels Lactic acidosis Exercise intolerance Short stature Hearing loss Diabetes Most often (80%) associated with the m.3243A > G variant in the MTTL1 gene encoding tRNALeu
MERRF Myoclonic epilepsy with ragged red fibers	Myoclonic epilepsy, including other types of seizures Symptoms in childhood after a period of normal development Cerebellar ataxia, myopathy, hearing loss, optic atrophy, cardiac arrhythmias, dementia Muscle biopsy (Gomori trichrome stain) Shows pathognomonic, ragged muscle fibers with red granules under the sarco-lemma—ragged red fibers Most often (80%) associated with the m.8344A > G mutation in the MTTK gene encoding tRNALeu
Kearns–Sayre syndrome	Age of onset < 20 years Retinitis pigmentosa (twilight vision disturbances, narrowing of the visual field to telescope vision, photophobia) Progressive external ophthalmoplegia (ptosis, limited eye movement) Cardiac conduction disorders (AV blocks) Hearing loss, diabetes, hypoparathyroidism, growth hormone deficiency, elevated CSF protein levels, cerebral folate deficiency (leukoencephalopathy) mtDNA deletions (most often m.8470_13446del4977)
MNGIE Mitochondrial neurogastrointestinal encephalomyopathy	Symptoms onset: childhood, adolescence/young adulthood (typical), adult-hood (late onset, >40 years) GI symptoms/signs: sub-occlusive episodes, nausea, vomiting, early satiety, severe abdominal pain, abdominal distension, dysphagia, constipation and diarrhea, acute peritonitis due to small bowel perforation Unexplained weight loss, thinness, cachexia Radiological GI signs: small bowel diverticulosis, GI dilation (e.g., gastric or intestinal dilation) Neurological symptoms/signs: chronic progressive external ophthalmoplegia (CPEO), ptosis, peripheral neuropathy, hearing loss Neuroradiological signs: leukoencephalopathy without other neuroradiological abnormalities Metabolic alterations: liver steatosis evolving in cirrhosis, pancreatitis, early-onset diabetes mellitus, increased triglyceride levels, elevated plasma lactate

**Table 7:** Clinical features of HPP in children.

Type of HPP	Clinical Features
Lethal perinatal HPP	polyhydramnios, bowed and short long bones, low or absent skeletal mineralization, caput membranaceum, hypoplastic thoracic cage; respiratory distress due to pulmonary hypoplasia, tracheomalacia, chest deformity and profound muscular weakness; pyridoxine-dependent seizures; increased intracranial pressure (papilledema, vomiting); hypercalcemia and hypercalciuria and sometimes nephrocalcinosis
Benign perinatal HPP	limb shortening with bowing of the long bones showing spontaneous improvement in the last gestational trimester or at birth
Infantile HPP	newborns appear healthy at the time of birth; failure to thrive, poor feeding, muscular weakness, developmental delay, signs resembling rickets, i.e., wide fontanelles and rachitic deformities; respiratory failure due to pulmonary hypoplasia, small thorax, gracile bones, recurrent fractures, and tracheomalacia; hypercalcemia and hypercalciuria and sometimes nephrocalcinosis; craniosynostosis and intracranial hypertension; pyridoxine-dependent seizures; untreated patients with infantile HPP have 50% mortality in the first year of life
Childhood HPP Mild form	minor or no symptoms; early tooth loss (premature painless exfoliation of one or more deciduous teeth with intact roots before age 5 years); radiographic skeletal changes are very subtle, e.g., low bone mass
Childhood HPP Severe form	premature tooth loss; skeletal pain; muscle weakness (delayed walking, waddling gait, difficulty in climbing stairs); skeletal deformities—pectus excavatum, craniosynostosis, scoliosis and deformed long bones (slow-healing recurrent fractures; genu varum or genu valgum, swollen wrists (metaphyseal flaring))
OdontoHPP	early loss of deciduous (before 3–5 years of age) and permanent teeth without signs of periodontal inflammation; defects in the shape, structure, and color of teeth, hypoplasia of enamel and dentine, thin dentinal walls, wide pulp chambers, thin and short roots, and dental caries

**Table 8:** IMDs with lactic acidosis [75,77].

1. Defects of pyruvate metabolism Pyruvate dehydrogenase deficiency Pyruvate carboxylase deficiency
2. Defects of NADH oxidation Defects of the mitochondrial electron transfer chain
3. Gluconeogenesis disorders/glycogen storage disorders Glucose-6-phosphatase deficiency (GSD I) Fructose-1,6-bisphosphatase deficiency Phosphoenolpyruvate carboxykinase deficiency Glycogen debrancher deficiency (GSD III) Glycogen synthase deficiency (GSD 0)
4. Defects of fatty acid oxidation
5. Defects of biotin metabolism Biotinidase deficiency Holocarboxylase synthase deficiency
6. Defects of organic acid metabolism Propionic acidosis Methylmalonic acidosis
7. Other Hereditary fructose intolerance

**Table 9:** Etiology of hyperammonemia [83,84].

**IMDs**
Urea cycle defects Organic acidurias Fatty acid oxidation disorders Hypoglycemia–hyperammonemia syndrome
**Secondary**
Liver failure Portosystemic shunt Medications: valproate, L-asparaginase Physical exertion (e.g., seizures, respiratory failure)
**False-positive results**
Blood squeezing Sample hemolysis Long-term blood sample storage

**Table 10:** Clinical presentation of UCDs [80,81].

Acute symptoms/clinical features
Consciousness disturbances (ranging from drowsiness to coma) Seizures Vomiting Encephalopathy Acute liver failure, coagulopathy (especially in OTCD and HHH) Circulatory failure, multi-organ failure Psychiatric symptoms (hallucinations, mania, psychosis, emotional. or personality disorders) In newborns: sepsis-like appearance, body temperature instability, hyperventilation
Chronic symptoms/clinical features
Recurrent symptoms Exacerbation following infections, excessive protein intake, or fasting Protein aversion Consciousness disturbances Cerebellar symptoms (tremor, ataxia, dysarthria) Headaches (migraine-like) Learning difficulties, cognitive impairment Epilepsy Progressive spastic diplegia or tetraplegia (described as ARG1D, HHH syndrome) Recurrent abdominal pain and vomiting Poor physical development (underweight and height) Elevated aminotransferase levels Psychiatric symptoms: hyperactivity, mood swings, behavioral changes, aggression Autistic features Trichorrhesis nodosa (ASLD)
Factors that may cause decompensation
Infection, especially with fever Excessive protein intake or fasting Gastrointestinal bleeding Prolonged, intense physical exertion Surgery under general anesthesia Medications: valproate, L-asparaginase, high-dose glucocorticosteroids, topiramate, carbamazepine, phenobarbital, phenytoin, primidone, furosemide, hydrochlorothiazide, salicylates

**Table 12:** When to suspect IMD—red flags.

**Correlations of Clinical and Biochemical Features Suggestive for IMD**
Megaloblastic anemia + feeding difficulties/failure to thrive + developmental delay + seizures Megaloblastic anemia + renal manifestations (haemolytic uraemic syndrome) Hepatosplenomegaly (suggestive for storage) + thrombocytopenia Acute (hyperammonemic) encephalopathy Neurological deterioration ± lactic acidosis ± hyperammonaemia ± hypoglycemia Hepatopathy (elevated serum transaminases, prolonged INR) + myopathy (elevated CK activity, cardiomyopathy) + hypoketotic hypoglycemia ± lactic acidosis ± hyperammonaemia Early-onset encephalopathy + lactic acidosis ± brain malformations Acute/chronic encephalopathy + neutropenia/pancytopenia ± cardiomyopathy Acute/chronic encephalopathy + neutropenia/pancytopenia ± chronic renal failure Hepatomegaly + elevated serum transaminases + hypercholesterolemia ± hypoglycemia Recurrent hypoglycemia + hepatomegaly Hepatomegaly + elevated serum transaminases + tubulopathy Fasting hypoglycemia + hepatomegaly + elevated serum transaminases ± neutropenia Acute liver failure (especially in early childhood) Recurrent myalgia (exercise-induced) + rhabdomyolisis Permanently elevated CK activity + hepatomegaly + elevated serum transaminases + hypoglycemia Hyperuricemia + developmental delay/intellectual disability + movement disorders Hypouricemia + neonatal epileptic encephalopathy Hypouricemia + urolithiasis Neurological manifestation (ataxia, peripheral neuropathy) + hypocholesterolemia Recurrent pancreatitis + hypertriglyceridemia Elevated ALP activity + intellectual disability ± epilepsy Decreased ALP activity + rickets-like features

## Data Availability

The original contributions presented in this study are included in the article/Supplementary Material. Further inquiries can be directed to the corresponding author.

## Supplementary Materials

The following supporting information can be downloaded at: https://www.mdpi.com/article/10.3390/diagnostics15212806/s1, Table S1. Hepatic phenotype of mitochondrial hepatopathies related to OXPHOS deficiencies. Table S2. Causes of serum CK elevation [37]. Table S3. Clinical symptoms of mitochondrial disorders [44,45,46]. Table S4. Causes of low ALP activity [57,58,59]. Table S5. Various causes of elevated serum uric acid [61]. Table S6. Various causes of elevated serum uric acid [61]. Figure S1. Schematic presentation of purine metabolism.

## References

[1] Ferreira C.R., van Karnebeek C.D.M. (2019). Inborn Errors of Metabolism. Handbook of Clinical Neurology.

[2] Saudubray J.M., Garcia-Cazorla À. (2018). Inborn Errors of Metabolism Overview: Pathophysiology, Manifestations, Evaluation, and Management. Pediatr. Clin. N. Am..

[3] Vernon H.J. (2015). Inborn Errors of Metabolism: Advances in Diagnosis and Therapy. JAMA Pediatr..

[4] Ferreira C.R., van Karnebeek C.D.M., Vockley J., Blau N. (2019). A proposed nosology of inborn errors of metabolism. Genet. Med..

[5] Ferreira C.R., Rahman S., Keller M., Zschocke J., ICIMD Advisory Group (2021). An international classification of inherited metabolic disorders (ICIMD). J. Inherit. Metab. Dis..

[6] Lalonde E., Rentas S., Lin F., Dulik M.C., Skraban C.M., Spinner N.B. (2020). Genomic Diagnosis for Pediatric Disorders: Revolution and Evolution. Front. Pediatr..

[7] Moutapam-Ngamby-Adriaansen Y., Maillot F., Labarthe F., Lioger B. (2024). Blood cytopenias as manifestations of inherited metabolic diseases: A narrative review. Orphanet J. Rare Dis..

[8] Cario H., Smith D.E., Blom H., Blau N., Bode H., Holzmann K., Pannicke U., Hopfner K.P., Rump E.M., Ayric Z. (2011). Dihydrofolate reductase deficiency due to a homozygous DHFR mutation causes megaloblastic anemia and cerebral folate deficiency leading to severe neurologic disease. Am. J. Hum. Genet..

[9] Hilton J.F., Christensen K.E., Watkins D., Raby B.A., Renaud Y., de la Luna S., Estivill X., MacKenzie R.E., Hudson T.J., Rosenblatt D.S. (2003). The molecular basis of glutamate formiminotransferase deficiency. Hum. Mutat..

[10] Fujiwara T., Harigae H. (2013). Pathophysiology and genetic mutations in congenital sideroblastic anemia. Pediatr. Int..

[11] Lecha M., Puy H., Deybach J.C. (2009). Erythropoietic protoporphyria. Orphanet J. Rare Dis..

[12] Balwani M. (2019). Erythropoietic Protoporphyria and X-Linked Protoporphyria: Pathophysiology, genetics, clinical manifestations, and management. Mol. Genet. Metab..

[13] Szlendak U., Bykowska K., Lipniacka A. (2016). Clinical, Biochemical and Molecular Characteristics of the Main Types of Porphyria. Adv. Clin. Exp. Med..

[14] To-Figueras J., Erwin A.L., Aguilera P., Millet O., Desnick R.J. (2024). Congenital erythropoietic porphyria. Liver Int..

[15] Yoshimi A., Ishikawa K., Niemeyer C., Grünert S.C. (2022). Pearson syndrome: A multisystem mitochondrial disease with bone marrow failure. Orphanet J. Rare Dis..

[16] Riley L.G., Heeney M.M., Rudinger-Thirion J., Frugier M., Campagna D.R., Zhou R., Hale G.A., Hilliard L.M., Kaplan J.A., Kwiatkowski J.L. (2018). The phenotypic spectrum of germline *YARS2* variants: From isolated sideroblastic anemia to mitochondrial myopathy, lactic acidosis and sideroblastic anemia 2. Haematologica.

[17] Lipiński P., Tylki-Szymańska A. (2024). The Liver and Lysosomal Storage Diseases: From Pathophysiology to Clinical Presentation, Diagnostics, and Treatment. Diagnostics.

[18] Stirnemann J., Belmatoug N., Camou F., Serratrice C., Froissart R., Caillaud C., Levade T., Astudillo L., Serratrice J., Brassier A. (2017). A Review of Gaucher Disease Pathophysiology, Clinical Presentation and Treatments. Int. J. Mol. Sci..

[19] Steward C.G., Groves S.J., Taylor C.T., Maisenbacher M.K., Versluys B., Newbury-Ecob R.A., Ozsahin H., Damin M.K., Bowen V.M., McCurdy K.R. (2019). Neutropenia in Barth syndrome: Characteristics, risks, and management. Curr. Opin. Hematol..

[20] Grünert S.C., Derks T.G.J., Mundy H., Dalton R.N., Donadieu J., Hofbauer P., Jones N., Uçar S.K., LaFreniere J., Contreras E.L. (2024). Treatment recommendations for glycogen storage disease type IB- associated neutropenia and neutrophil dysfunction with empagliflozin: Consensus from an international workshop. Mol. Genet. Metab..

[21] Kishnani P.S., Austin S.L., Abdenur J.E., Arn P., Bali D.S., Boney A., Chung W.K., Dagli A.I., Dale D., Koeberl D. (2014). Diagnosis and management of glycogen storage disease type I: A practice guideline of the American College of Medical Genetics and Genomics. Genet. Med..

[22] Pascoal C., Francisco R., Mexia P., Luis Pereira B., Granjo P., Coelho H., Barbosa M., Dos Reis Ferreira V., Videira P.A. (2024). Revisiting the immunopathology of congenital disorders of glycosylation: An updated review. Front. Immunol..

[23] Zhang Y., Yu X., Ichikawa M., Lyons J.J., Datta S., Lamborn I.T., Jing H., Kim E.S., Biancalana M., Wolfe L.A. (2014). Autosomal recessive phosphoglucomutase 3 (PGM3) mutations link glycosylation defects to atopy, immune deficiency, autoimmunity, and neurocognitive impairment. J. Allergy Clin. Immunol..

[24] Stray-Pedersen A., Backe P.H., Sorte H.S., Mørkrid L., Chokshi N.Y., Erichsen H.C., Gambin T., Elgstøen K.B., Bjørås M., Wlodarski M.W. (2014). PGM3 mutations cause a congenital disorder of glycosylation with severe immunodeficiency and skeletal dysplasia. Am. J. Hum. Genet..

[25] Forny P., Hörster F., Ballhausen D., Chakrapani A., Chapman K.A., Dionisi-Vici C., Dixon M., Grünert S.C., Grunewald S., Haliloglu G. (2021). Guidelines for the diagnosis and management of methylmalonic acidaemia and propionic acidaemia: First revision. J. Inherit. Metab. Dis..

[26] Ferreira C.R., Cassiman D., Blau N. (2019). Clinical and biochemical footprints of inherited metabolic diseases. II. Metabolic liver diseases. Mol. Genet. Metab..

[27] Seker Yilmaz B., Baruteau J., Rahim A.A., Gissen P. (2020). Clinical and Molecular Features of Early Infantile Niemann Pick Type C Disease. Int. J. Mol. Sci..

[28] Klouwer F.C., Berendse K., Ferdinandusse S., Wanders R.J., Engelen M., Poll-The B.T. (2015). Zellweger spectrum disorders: Clinical overview and management approach. Orphanet J. Rare Dis..

[29] Heubi J.E., Setchell K.D., Bove K.E. (2007). Inborn errors of bile acid metabolism. Semin. Liver Dis..

[30] Spiekerkoetter U. (2010). Mitochondrial fatty acid oxidation disorders: Clinical presentation of long-chain fatty acid oxidation defects before and after newborn screening. J. Inherit. Metab. Dis..

[31] Guerra I.M.S., Ferreira H.B., Melo T., Rocha H., Moreira S., Diogo L., Domingues M.R., Moreira A.S.P. (2022). Mitochondrial Fatty Acid β-Oxidation Disorders: From Disease to Lipidomic Studies-A Critical Review. Int. J. Mol. Sci..

[32] Knottnerus S.J.G., Bleeker J.C., Wüst R.C.I., Ferdinandusse S., IJlst L., Wijburg F.A., Wanders R.J.A., Visser G., Houtkooper R.H. (2018). Disorders of mitochondrial long-chain fatty acid oxidation and the carnitine shuttle. Rev. Endocr. Metab. Disord..

[33] Lee W.S., Sokol R.J. (2007). Mitochondrial hepatopathies: Advances in genetics and pathogenesis. Hepatology.

[34] Gopan A., Sarma M.S. (2021). Mitochondrial hepatopathy: Respiratory chain disorders-‘breathing in and out of the liver’. World J. Hepatol..

[35] Al-Hussaini A., Faqeih E., El-Hattab A.W., Alfadhel M., Asery A., Alsaleem B., Bakhsh E., Ali A., Alasmari A., Lone K. (2014). Clinical and molecular characteristics of mitochondrial DNA depletion syndrome associated with neonatal cholestasis and liver failure. J. Pediatr..

[36] Shimura M., Kuranobu N., Ogawa-Tominaga M., Akiyama N., Sugiyama Y., Ebihara T., Fushimi T., Ichimoto K., Matsunaga A., Tsuruoka T. (2020). Clinical and molecular basis of hepatocerebral mitochondrial DNA depletion syndrome in Japan: Evaluation of outcomes after liver transplantation. Orphanet J. Rare Dis..

[37] Aleksovska K., Kyriakides T., Angelini C., Argov Z., Claeys K.G., de Visser M., Filosto M., Jovanovic I., Kostera-Pruszczyk A., Molnar M.J. (2025). What Are the Normal Serum Creatine Kinase Values for Skeletal Muscle? A Worldwide Systematic Review. Eur. J. Neurol..

[38] Urtizberea J.A., Severa G., Malfatti E. (2023). Metabolic Myopathies in the Era of Next-Generation Sequencing. Genes.

[39] Lilleker J.B., Keh Y.S., Roncaroli F., Sharma R., Roberts M. (2018). Metabolic myopathies: A practical approach. Pract. Neurol..

[40] Farrar M.A., Kariyawasam D., Grattan S., Bayley K., Davis M., Holland S., Waddel L.B., Jones K., Lorentzos M., Ravine A. (2023). Newborn Screening for the Diagnosis and Treatment of Duchenne Muscular Dystrophy. J. Neuromuscul. Dis..

[41] Tarnopolsky M.A. (2018). Myopathies Related to Glycogen Metabolism Disorders. Neurotherapeutics.

[42] Gümüş E., Özen H. (2023). Glycogen storage diseases: An update. World J. Gastroenterol..

[43] Kishnani P.S., Steiner R.D., Bali D., Berger K., Byrne B.J., Case L.E., Crowley J.F., Downs S., Howell R.R., Kravitz R.M. (2006). Pompe disease diagnosis and management guideline. Genet. Med..

[44] Olimpio C., Tiet M.Y., Horvath R. (2021). Primary mitochondrial myopathies in childhood. Neuromuscul. Disord..

[45] Bangeas A., Poulidou V., Liampas I., Marogianni C., Aloizou A.M., Tsouris Z., Sgantzos M., Arnaoutoglou M., Bogdanos D.P., Dardiotis E. (2025). Advances in Management of Mitochondrial Myopathies. Int. J. Mol. Sci..

[46] Ahmed S.T., Craven L., Russell O.M., Turnbull D.M., Vincent A.E. (2018). Diagnosis and Treatment of Mitochondrial Myopathies. Neurotherapeutics.

[47] Cannalire G., Pilloni S., Esposito S., Biasucci G., Di Franco A., Street M.E. (2023). Alkaline phosphatase in clinical practice in childhood: Focus on rickets. Front. Endocrinol..

[48] Strauch J.M., Vogel M., Meigen C., Ceglarek U., Kratzsch J., Willenberg A., Kiess W. (2023). Pediatric reference values of alkaline phosphatase: Analysis from a German population-based cohort and influence of anthropometric and blood parameters. Bone.

[49] Colantonio D.A., Kyriakopoulou L., Chan M.K., Daly C.H., Brinc D., Venner A.A., Pasic M.D., Armbruster D., Adeli K. (2012). Closing the gaps in pediatric laboratory reference intervals: A CALIPER database of 40 biochemical markers in a healthy and multiethnic population of children. Clin. Chem..

[50] Ridefelt P., Gustafsson J., Aldrimer M., Hellberg D. (2014). Alkaline phosphatase in healthy children: Reference intervals and prevalence of elevated levels. Horm. Res. Pediatr..

[51] Horn D., Schottmann G., Meinecke P. (2010). Hyperphosphatasia with mental retardation, brachytelephalangy, and a distinct facial gestalt: Delineation of a recognizable syndrome. Eur. J. Med. Genet..

[52] Carmody L.C., Blau H., Danis D., Zhang X.A., Gourdine J.P., Vasilevsky N., Krawitz P., Thompson M.D., Robinson P.N. (2020). Significantly different clinical phenotypes associated with mutations in synthesis and transamidase+remodeling glycosylphosphatidylinositol (GPI)-anchor biosynthesis genes. Orphanet J. Rare Dis..

[53] Hutny M., Lipinski P., Jezela-Stanek A. (2023). Characteristics of Neuroimaging and Behavioural Phenotype in Polish Patients with PIGV-CDG-An Observational Study and Literature Review. Genes.

[54] Messina M., Manea E., Cullup T., Tuschl K., Batzios S. (2022). Hyperphosphatasia with mental retardation syndrome 3: Cerebrospinal fluid abnormalities and correction with pyridoxine and Folinic acid. JIMD Rep..

[55] Shkalim Zemer V., Hoshen M., Levinsky Y., Richenberg Y., Yosef N., Oberman B., Cohen M., Cohen A.H. (2023). Benign transient hyperphosphatasemia in infants and children: A retrospective database study. Eur. J. Pediatr..

[56] Huh S.Y., Feldman H.A., Cox J.E., Gordon C.M. (2009). Prevalence of transient hyperphosphatasemia among healthy infants and toddlers. Pediatrics.

[57] Whyte M.P. (2016). Hypophosphatasia—Aetiology, nosology, pathogenesis, diagnosis and treatment. Nat. Rev. Endocrinol..

[58] Mornet E. (2018). Hypophosphatasia. Metabolism.

[59] Bianchi M.L. (2015). Hypophosphatasia: An overview of the disease and its treatment. Osteoporos. Int..

[60] Fumagalli M., Lecca D., Abbracchio M.P., Ceruti S. (2017). Pathophysiological Role of Purines and Pyrimidines in Neurodevelopment: Unveiling New Pharmacological Approaches to Congenital Brain Diseases. Front. Pharmacol..

[61] Fathallah-Shaykh S.A., Cramer M.T. (2014). Uric acid and the kidney. Pediatr. Nephrol..

[62] Simoni R.E., Gomes L.N., Scalco F.B., Oliveira C.P., Aquino Neto F.R., de Oliveira M.L. (2007). Uric acid changes in urine and plasma: An effective tool in screening for purine inborn errors of metabolism and other pathological conditions. J. Inherit. Metab. Dis..

[63] Torres R.J., Puig J.G. (2007). Hypoxanthine-guanine phosophoribosyltransferase (HPRT) deficiency: Lesch-Nyhan syndrome. Orphanet J. Rare Dis..

[64] Torun B., Bilgin A., Orhan D., Gocmen R., Kılıc S.S., Kuskonmaz B., Cetinkaya D., Tezcan I., Cagdas D. (2022). Combined immunodeficiency due to purine nucleoside phosphorylase deficiency: Outcome of three patients. Eur. J. Med. Genet..

[65] Peretz H., Lagziel A., Bittner F., Kabha M., Shtauber-Naamati M., Zhuravel V., Usher S., Rump S., Wollers S., Bork B. (2021). Classical Xanthinuria in Nine Israeli Families and Two Isolated Cases from Germany: Molecular, Biochemical and Population Genetics Aspects. Biomedicines.

[66] Cho S.K., Schwarz G., Tasic V., Křížková M., Krijt J., Roeper J., Honzík T., Šebesta I., Kožich V., Šaligová J. (2025). A prevalent MOCS2 variant in the Roma population is associated with a novel mild form of molybdenum cofactor deficiency. Eur. J. Pediatr..

[67] Dąbrowska-Leonik N., Piątosa B., Słomińska E., Bohynikova N., Bernat-Sitarz K., Bernatowska E., Wolska-Kuśnierz B., Kałwak K., Kołtan S., Dąbrowska A. (2023). National experience with adenosine deaminase deficiency related SCID in Polish children. Front. Immunol..

[68] Jurecka A., Zikanova M., Kmoch S., Tylki-Szymańska A. (2015). Adenylosuccinate lyase deficiency. J. Inherit. Metab. Dis..

[69] Quarta A., Iannucci D., Guarino M., Blasetti A., Chiarelli F. (2023). Hypoglycemia in Children: Major Endocrine-Metabolic Causes and Novel Therapeutic Perspectives. Nutrients.

[70] Casertano A., Rossi A., Fecarotta S., Rosanio F.M., Moracas C., Di Candia F., Parenti G., Franzese A., Mozzillo E. (2021). An Overview of Hypoglycemia in Children Including a Comprehensive Practical Diagnostic Flowchart for Clinical Use. Front. Endocrinol..

[71] Hannah W.B., Derks T.G.J., Drumm M.L., Grünert S.C., Kishnani P.S., Vissing J. (2023). Glycogen storage diseases. Nat. Rev. Dis. Primers.

[72] Kishnani P.S., Goldstein J., Austin S.L., Arn P., Bachrach B., Bali D.S., Chung W.K., El-Gharbawy A., Brown L.M., Kahler S. (2019). Diagnosis and management of glycogen storage diseases type VI and IX: A clinical practice resource of the American College of Medical Genetics and Genomics (ACMG). Genet. Med..

[73] Koch R.L., Soler-Alfonso C., Kiely B.T., Asai A., Smith A.L., Bali D.S., Kang P.B., Landstrom A.P., Akman H.O., Burrow T.A. (2023). Diagnosis and management of glycogen storage disease type IV, including adult polyglucosan body disease: A clinical practice resource. Mol. Genet. Metab..

[74] Berling É., Laforêt P., Wahbi K., Labrune P., Petit F., Ronzitti G., O’Brien A. (2021). Narrative review of glycogen storage disorder type III with a focus on neuromuscular, cardiac and therapeutic aspects. J. Inherit. Metab. Dis..

[75] Belu A., Filip N., Trandafir L.M., Spoială E.L., Țarcă E., Zamosteanu D., Ghiga G., Bernic J., Jehac A., Cojocaru E. (2025). Lactate, an Essential Metabolic Marker in the Diagnosis and Management of Pediatric Conditions. Diagnostics.

[76] Schumann A., Schultheiss U.T., Ferreira C.R., Blau N. (2023). Clinical and biochemical footprints of inherited metabolic diseases. XIV. Metabolic kidney diseases. Mol. Genet. Metab..

[77] Müller J., Radej J., Horak J., Karvunidis T., Valesova L., Kriz M., Matejovic M. (2023). Lactate: The Fallacy of Oversimplification. Biomedicines.

[78] Shayota Brian J. (2024). Biomarkers of mitochondrial disorders. Neurotherapeutics.

[79] Ganetzky R., McCormick E.M., Falk M.J., Adam M.P., Feldman J., Mirzaa G.M., Pagon R.A., Wallace S.E., Amemiya A. (2021). Primary Pyruvate Dehydrogenase Complex Deficiency Overview. GeneReviews^®^.

[80] Savvidou A., Ivarsson L., Naess K., Eklund E.A., Lundgren J., Dahlin M., Frithiof D., Sofou K., Darin N. (2022). Novel imaging findings in pyruvate dehydrogenase complex (PDHc) deficiency-Results from a nationwide population-based study. J. Inherit. Metab. Dis..

[81] Weinstein D.A., Correia C.E., Saunders A.C., Wolfsdorf J.I. (2006). Hepatic glycogen synthase deficiency: An infrequently recognized cause of ketotic hypoglycemia. Mol. Genet. Metab..

[82] Häberle J. (2011). Clinical practice: The management of hyperammonemia. Eur. J. Pediatr..

[83] Häberle J., Burlina A., Chakrapani A., Dixon M., Karall D., Lindner M., Mandel H., Martinelli D., Pintos-Morell G., Santer R. (2019). Suggested guidelines for the diagnosis and management of urea cycle disorders: First revision. J. Inherit. Metab. Dis..

[84] Goldberg A.C., Hopkins P.N., Toth P.P., Ballantyne C.M., Rader D.J., Robinson J.G., Daniels S.R., Gidding S.S., de Ferranti S.D., Ito M.K. (2011). Familial hypercholesterolemia: Screening, diagnosis and management of pediatric and adult patients. Clinical guidance from the National Lipid Association Expert Panel on Familial Hypercholesterolemia. J. Clin. Lipidol..

[85] Nielsen S.T., Lytsen R.M., Strandkjaer N., Rasmussen I.J., Sillesen A.-S., Vøgg R.O.B., Raja A.A., Nordestgaard B.G., Kamstrup P.R., Iversen K. (2023). Significance of lipids, lipoproteins, and apolipoproteins during the first 14–16 months of life. Eur. Heart J..

[86] Myśliwiec M., Bandura M., Wołoszyn-Durkiewicz A., Hennig M., Walczak M., Peregud-Pogorzelski J., Sykut-Cegielska J., Miszczak-Knecht M., Chlebus K., Wasąg B. (2024). 2024 Polish recommendations for the management of familial hypercholesterolemia in children and adolescents. Arch. Med. Sci..

[87] Shah A.S., Wilson D.P., Feingold K.R., Ahmed S.F., Anawalt B., Blackman M.R., Boyce A., Chrousos G., Corpas E., de Herder W.W., Dhatariya K., Dungan K. (2023). Genetic Disorders Causing Hypertriglycerydemia in Children and Adolescents.

[88] Moutzouri E., Elisaf M., Liberpoulos E.N. (2011). Hypocholesterolemia. Curr. Vasc. Pharmacol..

[89] Groselj U., Kafol J., Molk N., Sedej K., Mlinaric M., Sikonja J., Sustar U., Kern B.C., Kovac J., Battelino T. (2025). Prevalence, genetic variants, and clinical implications of hypocholesterolemia in children. Atherosclerosis.

