# Supplementary material for: The Usefulness of Basic Laboratory Analyses in Diagnostics of Inherited Metabolic Diseases in Children
Source: Diagnostics (Basel). 2025 Nov 5;15(21):2806. doi: 10.3390/diagnostics15212806 (PMC12610540; doi:10.3390/diagnostics15212806)
Supplement: Supplementary file 1 [file diagnostics-15-02806-s001.zip › Suppl_Table_S1.pdf]

Gene<br>Disease<br>OMIM number	Liver enlargement	Elevated liver transaminases	Cholestasis	Acute liver failure	Liver steatosis	Liver fibrosis /cirrhodid	Liver carcinoma	**Other**
Mutations in nDNA genes causing mtDNA depletion								
DGUOK<br>Mitochondrial DNA depletion syndrome 3 (hepatocerebral type)<br>#251880	+	+	+	+	+	+	HCC	Hypotonia, nystagmus, microcephaly, delayed psychomotor development
POLG1<br>Mitochondrial DNA depletion syndrome 4A (Alpers type)<br>#203700	+	+	+	+	+	+		Drug-resistant epilepsy, psychomotor regression, cortical blindness
MPV17<br>Mitochondrial DNA depletion syndrome 6 (hepatocerebral type)<br>#256810	+	+	+	+	+	+	HCC	Hypotonia, psychomotor regression, ataxia, peripheral neuropathy
TWNK (C10orf2)<br>Mitochondrial DNA depletion syndrome 7 (hepatocerebral type)<br>#271245	+	+	+	+	+	+		Hypotonia, psychomotor regression, ataxia, peripheral neuropathy, epilepsy, ophthalmoplegia
SUCLG1	+	+		+				Myopathy (severe hypotonia), psychomotor regression, MRI resembling

Mitochondrial DNA depletion syndrome 9 (encephalomyopathic type with methylmalonic aciduria)<br><br>#245400								Leigh syndrome; Methylmalonic acidosis in urine organic acid profile (GC/MS)
Mutations in nDNA genes causing deficits in individual respiratory chain complexes								
BCS1L<br><br>GRACILE syndrome (*growth retardation, amino aciduria, cholestasis, iron overload, lactic acidosis, and early death*)<br><br>#603358	+	+	+		+			Complex III Deficiency<br><br>IUGR, aminoaciduria (Fanconi syndrome), hepatic iron accumulation;<br><br>In the Finnish population, the founder effect
SCO1<br><br>Mitochondrial complex IV deficiency, nuclear type 4<br><br>(# 619048)	+	+		+	+			Complex IV Deficiency<br><br>IUGR, hypotonia, psychomotor regression, hypertrophic cardiomyopathy
Mutations in nDNA genes encoding transcription or translation factors								
TRMU<br><br>Liver failure, transient infantile<br><br>(# 613070)	+	+	+	+	+	+		Disease resolution in patients who survived infantile liver failure.<br><br>Less frequently, hypertrophic cardiomyopathy, hypotension, and Leigh syndrome
GFM1	+	+		+		+		FGR, microcephaly, hypotonia, psychomotor regression,

Combined oxidative phosphorylation deficiency 1<br><br>(# 609060)								
MRPS16<br><br>Combined oxidative phosphorylation deficiency 2<br><br>(# 610498)	+	+		+	+	+		FGR, microcephaly, hypotonia, psychomotor regression, agenesis of the corpus callosum,
TSFM<br><br>Combined oxidative phosphorylation deficiency 3<br><br>#610505	+	+	+	+	+	+		FGR, hypotonia, psychomotor regression, rhabdomyolysis, hypertrophic cardiomyopathy, Leigh syndrome
TUFM<br><br>Combined oxidative phosphorylation deficiency 4<br><br>#610678	+	+	+	+	+	+		FGR, hypotonia, psychomotor regression,
