# Supplementary material for: The Usefulness of Basic Laboratory Analyses in Diagnostics of Inherited Metabolic Diseases in Children
Source: Diagnostics (Basel). 2025 Nov 5;15(21):2806. doi: 10.3390/diagnostics15212806 (PMC12610540; doi:10.3390/diagnostics15212806)
Supplement: Supplementary file 1 [file diagnostics-15-02806-s001.zip › Suppl_Table_S2.pdf]

Physical exertion
Surgery
Seizures
Infections, fever
Electrolyte disturbances
Celiac disease
Subclinical hypothyroidism
Systemic connective tissue diseases
Cancer
Pregnancy
Medications (statins, β-blockers, angiotensin receptor antagonists, immunosuppressants, antiretroviral drugs, neuroleptics)
Malignant hyperthermia
Muscular dystrophies (Duchenne and Becker muscular dystrophy, limb-girdle dystrophies, myotonic dystrophy)
Inflammatory myopathies (dermatomyositis, inclusion body myositis)
Metabolic myopathies (lipid myopathies, mitochondrial myopathies, Pompe disease)

Supplementary Table S2. Causes of serum CK elevation [37].
