# Supplementary material for: The Usefulness of Basic Laboratory Analyses in Diagnostics of Inherited Metabolic Diseases in Children
Source: Diagnostics (Basel). 2025 Nov 5;15(21):2806. doi: 10.3390/diagnostics15212806 (PMC12610540; doi:10.3390/diagnostics15212806)
Supplement: Supplementary file 1 [file diagnostics-15-02806-s001.zip › Suppl_Table_S3.pdf]

Muscular System	Ptosis, external ophthalmoplegia, myalgia, limb-girdle muscle weakness, exercise intolerance
Central Nervous System	Stroke-like episodes, seizures, mental retardation, ataxia, headaches
Peripheral Nervous System	Polyneuropathy, orthostatic hypotension
Ophthalmologic Symptoms	Optic Nerve Atrophy, Retinitis Pigmentosa
Audiologic Symptoms	Sensorineural Hearing Loss
Cardiac Symptoms	Cardiomyopathy, conduction disturbances
Endocrine Symptoms	Hypothyroidism, hypoparathyroidism, adrenal insufficiency, diabetes, diabetes insipidus
Gastrointestinal Symptoms	Gastrointestinal motility disorders, liver damage
Hematological Symptoms	Anemia (sideroblastic), neutropenia, pancytopenia

Supplementary Table S3. Clinical symptoms of mitochondrial disorders [44-46].
