# Supplementary material for: The Usefulness of Basic Laboratory Analyses in Diagnostics of Inherited Metabolic Diseases in Children
Source: Diagnostics (Basel). 2025 Nov 5;15(21):2806. doi: 10.3390/diagnostics15212806 (PMC12610540; doi:10.3390/diagnostics15212806)
Supplement: Supplementary file 1 [file diagnostics-15-02806-s001.zip › Suppl_Table_S4.pdf]

cleidocranial dysplasia
osteogenesis imperfecta type II
hypothyroidism
Cushing’s syndrome
milk-alkali syndrome
celiac disease
vitamin C or D deficiency
zinc or magnesium deficiency
malnutrition, starvation
severe anaemia
treatment with bisphosphonates, glucocorticosteroids, densoumab
inadequate sample collection technique (oxalate, EDTA)
blood or plasma transfusion

Supplementary Table S4. Causes of low ALP activity [57-59].
