# Supplementary material for: The Usefulness of Basic Laboratory Analyses in Diagnostics of Inherited Metabolic Diseases in Children
Source: Diagnostics (Basel). 2025 Nov 5;15(21):2806. doi: 10.3390/diagnostics15212806 (PMC12610540; doi:10.3390/diagnostics15212806)
Supplement: Supplementary file 1 [file diagnostics-15-02806-s001.zip › Suppl_Table_S5.pdf]

UA overproduction	Paget’s bone disease<br>Psoriasis<br>Hemolytic anaemia<br>Myelo- and lymphoproliferative diseases; other malignancies<br>Tissue hypoxia<br>Rhabdomyolysis<br>Cytotoxic agents, cyclophosphamide<br>Pancreatic extract (high-dose therapy for cystic fibrosis)<br>Excessive dietary purine ingestion<br>Lesch-Nyhan disease<br>Kelly-Seegmiller syndrome<br>Phosphoribosyl pyrophosphate synthetase overactivity<br>hereditary fructose intolerance<br>fructose-1,6-biphosphatase deficiency<br>GSD types III, V and VII<br>MCAD (medium-chain acyl-CoA dehydrogenase) deficiency
UA underexcretion	Chronic renal failure<br>Dehydration; diabetes insipidus<br>Starvation ketoacidosis; diabetic ketoacidosis; lactic acidosis (tissue hypoxia)<br>Familial juvenile hyperuricemic nephropathy (FJHN)<br>Autosomal-dominant medullary cystic kidney disease (ADMCKD)

Supplementary Table S5. Various causes of elevated serum uric acid [61].
