# Supplementary material for: The Usefulness of Basic Laboratory Analyses in Diagnostics of Inherited Metabolic Diseases in Children
Source: Diagnostics (Basel). 2025 Nov 5;15(21):2806. doi: 10.3390/diagnostics15212806 (PMC12610540; doi:10.3390/diagnostics15212806)
Supplement: Supplementary file 1 [file diagnostics-15-02806-s001.zip › Suppl_Table_S6.pdf]

UA overexcretion	Acquired renal hypouricaemia–hyperuricosuria (drug-derived uricosuric agents)<br><br>Diabetes mellitus<br><br>Liver cirrhosis<br><br>Intracranial disease, SIADH syndrome – with hyponatremia<br><br>Drugs: probenecid and salicylates (high doses), sulfinpyrazone, ascorbic acid, oestrogen
UA underproduction	nucleoside phosphorylase (PNP) deficiency<br><br>xanthinurias I, II and III (molybdenum cofactor deficiency)

Supplementary Table S6. Various causes of elevated serum uric acid [61].
