# Supplementary figures and images for: The Usefulness of Basic Laboratory Analyses in Diagnostics of Inherited Metabolic Diseases in Children
Source: Diagnostics (Basel). 2025 Nov 5;15(21):2806. doi: 10.3390/diagnostics15212806 (PMC12610540; doi:10.3390/diagnostics15212806)

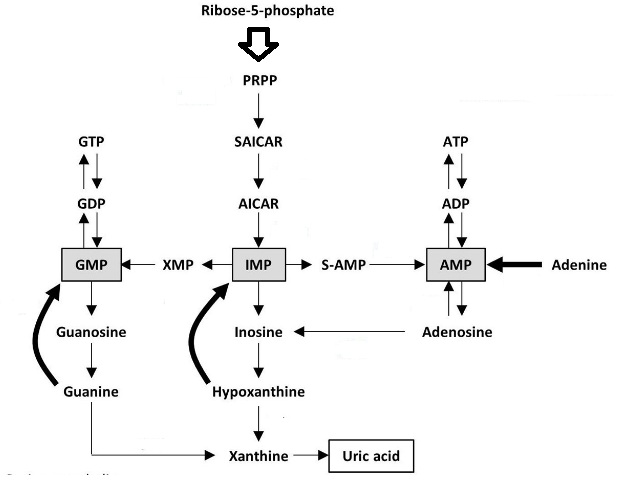

Supplement: Supplementary file 1 [file diagnostics-15-02806-s001.zip › Suppl_Figure_S1.jpg]
